# Crime, Education, and Insanity
*An Enquiry into the Extent and Causes of Juvenile Depravity. By Thomas Beggs. Gilpin.On Crime. By Mr. Flint. Gilpin.Moral and Educational Statistics. By J. Fletcher, Esq.


**Published:** 1852-04-01

**Authors:** 


／# THE JOURNAL

OF

### PSYCHOLOGICAL MEDICINE

AND

### MENTAL PATHOLOGY.

APRIL 1, 1852.

### Art. I.—CRIME, EDUCATION, AND INSANITY.*

Reason perplexes herself in vain for terms to define man in an irrational state. It is difficult to conceive of him as a mere animal; to divest him altogether of his intellectual attributes; to view him as a creation "in the form of God," and yet deprived of those faculties of memory, reflection, deduction, and calculation which essentially constitute the figurative resemblance: once degraded from humanity, his animal nature falls below the level of the brutes, for they never lose instinct but with life, and remain subject to self-preserving restraint. Language was not made for the portraiture of this anomalous condition; we are at a loss to express it even by paraphrase, or to idealize it with sufficient accuracy to convey the idea with perspicuity. So rare is the occurrence, that we have but one instance on scriptural record of a total separation of body and mind while life was still existent; and even this is only represented to us by one or two distinguishing traits that serve rather to indicate the grossness of the degradation than the new character of the subject of it. We learn that Nebuchadnezzar "was driven from men and did eat grass as oxen, and his body was wet with the dew of heaven, till his hairs were grown like eagle's feathers, and his nails like bird's claws." As a total prostration of humanity, the picture is complete; but we are left in ignorance of the extent to which human passions, human desires, or the appetites pecu-

* An Enquiry into the Extent and Causes of Juvenile Depravity. By Thomas Beggs. Gilpin.
On Crime. By Mr. Flint. Gilpin.
Moral and Educational Statistics. By J. Fletcher, Esq.

liar to humanity, were obliterated. As the severance of body and reason was total, and the degradation was designed for punishment, it is probable that the passions and desires remained, but without the power of gratification; unless we are to infer from the expression that his "understanding returned unto him," a temporary alienation of the soul from the body, during which the soul was in a state of suffering—an inference that may possibly be correct; for we are told that his punishment was to continue till he should "know that the Most High ruleth in the kingdom of men, and giveth it to whomsoever he will." This was a knowledge which an irrational being could not attain, as a conviction produced by suffering. It may be, therefore, that the intellectual and immaterial essence was in this case removed, and not that its functions were suspended; while the body it had inhabited, though not actually disengaged from its affections, was left wholly unfettered by its control, and unguided by its intelligence. It is idle to prosecute the speculative inquiry.

While we are at a loss to define intelligibly the status of human existence wholly apart from intellect or reason, the total severance of body and soul is an idea, if not intuitive, yet easily received, and confirmed by revelation as well as instilled by education; but the partial paralysis of the mind or soul, while yet in union with the body, affords a problem of which we can feel the difficulty, though we can find no words to convey it. Our passions are involved in a net of correlative intricacy; they are so interlaced with each other that a fibre cannot be wounded without affecting the sensibility of all. It is like a system of complicated machinery, in which the absence of a single screw may derange the whole, and put it out of working gear. The defect may be so trivial that the engineer cannot discover it: he examines the piston, the valves, the gauge, and all the apparatus; inspection can detect no flaw; there is no actual cessation of action, but it "does not work well." The engine is deranged, not incapacitated; his remedy is simple, though expensive; he takes it to pieces, and adjusts the parts again: but the derangement of the mind admits of no such process: we must bear with the bad working till close observation and long experience aid us in discovering the cause of failure; and if meanwhile we are called upon to explain the mystery, our only answer is, that our machinery is "not in working gear."

A closer examination into this web of human passions may be of service. It will not be disputed that there are to be found in every man, though modified in degree, the same dispositions to gratify, the same desires to be satisfied. The modification arises from difference of circumstances: it may be of constitution, of example, of opportunity, or otherwise; but the same nature that we all inherit, dictates to us the gratification of our animal wants: hunger, thirst, procreation, and self-protection, and, so far as consists with these, rest, are the desires which nature has implanted in us. Such is the impetuosity of these desires in animal nature not controlled by reason, that the brute creation will endanger life itself to gratify the three first, and are gifted with instinctive means to secure the others; but man, as an animal, is gregarious, and, as an intellectual animal, has convinced himself that society requires for its common safety the restraint of animal desires. To facilitate this self-restraint, the same intellectual faculty has suggested a gratification yet higher than that of animal passion, in self-complacency derived from the approbation of his fellows. Vanity will not persuade a man to die of hunger or thirst, but it will go far to keep him within the limits of due moderation, and to promote self-denial. His nature compels sociality; this is his animal instinct. His reason tells him that society has its laws, founded on what may be called a reciprocity of self-restraint. This is the position in which we find ourselves placed by nature as intellectual animals.

It is obvious that if there were an exact parity of circumstances, of motive, and of opportunity, we should be reduced to an instinctive state of existence. Like bees in a hive, we should do our duty as good citizens, each in his sphere, and nobody transgressing or falling short of his proper limit. We are not, however, mere animals, similarly circumstanced, but responsible beings; and to secure this responsibility, it has pleased the Creator to place us under variety of circumstances, affording variety of temptation. To some are given vivacity of temperament and robust health, tempting to sensuality; to some unbounded wealth, affording the solicitation of frequent opportunity; to others, and far more frequently, privation and distress that seduce into dishonest paths. Some are endowed with rank so exalted as to place them above the decencies of life; while others are depressed so low as not even to understand them: one can offend with impunity because he cannot lose caste; another has no caste to lose, and is, therefore, equally unrestrained. In all these varieties of position, the animal wants common to our race must be gratified alike; but the means of gratification being unequal, the inequality of means provokes a corresponding inequality of passion and of pursuit. The wealthy sensualist indulges in daily potations, large perhaps and enervating, but not intoxicating. The pauper sensualist lives like a hermit till Saturday night, and then gets drunk with gin. The rich debauchee, always gratified to satiety, degenerates into a saturated clod of earth; the needy drunkard alternates between the extreme of depression and the wild excitement of occasional inebriety; the former becomes morose, unreasonable, and tyrannical; the latter cunning, vindictive, and desponding; the first is uniformly indolent; the last, idle and energetic by turns. Or, perhaps, intemperance is not, in its grosser forms, the besetting sin in either case. Infirmity of body or early habit may prevent it; riches and opportunity may assault in another direction, and tempt their possessor to the purchase of power; power tends to oppression, oppression to resistance, and resistance to revenge. So, on the other hand, poverty may provoke to discontent, and discontent to rapacity and violence.

Nor is it only in the perversion of means to subserve licentious excess, but in the adaptation of them to legitimate gratification, that infinite scope is afforded for the range of passion; the trader begins in frugality and ends in avarice; the ingenuity of the mechanic often terminates in the gambling of the speculating patentee; the man of science pushes analysis and research to the verge of scepticism in revealed truth; perhaps (we say it doubtingly) the statesman may start in his career in a patriotic spirit, while the chances are a hundred to one that he closes it in political corruption, or selfish and unscrupulous ambition.

To check the vehemence of these passions, and correct their downward tendency, society has invented a double system of restraint; religion has imposed a third, more powerful than either; the decalogue has enumerated and prohibited specific offences of heinous guilt, under penalty of incurring the wrath of God; and the gospel has extended the prohibition and the penalty to the spirit as well as the letter of the offence. But we are not all religious; and therefore, to secure the common peace, society has promulgated her own codes in aid of the divine law: where passion is indulged to an extent that works actual injury or risk to others, legislation steps in to define the crime and visit it with appropriate punishment. This is one part of our restraining system; but it is clearly applicable only to cases where an evil intent is manifested by overt acts, and such as can be well defined not only by words but by their practical results. We can justly punish, because we can accurately describe, murder, theft, arson, forgery, and such like crimes; but, consistently with due regard to freedom, we cannot recognise constructive crime as a fair subject of penal enactment; in some instances we have gone to the very verge of discretion in the latitude of our legislative wisdom, but, in all free states, crime must be accurately defined, so as to admit of accurate proof, before it can be rendered penal.

Yet passion may be indulged to a very culpable excess, without transgressing the boundary that legislation has declared; to provide some restraint even in such cases, society has attached to this culpability what may be called a moral penalty, in the forfeiture of its good opinion; thus availing itself of a peculiarity of our nature which was, doubtless, implanted in us for good purposes, though often productive of the worst. It cannot be that men were created to live together, and yet to be indifferent to the esteem and respect of their fellow-creatures: to set this up as a paramount motive, would be to disobey the command to fear God rather than man; to disregard it as a secondary motive, is to despise the example of Christ Himself, who, as He grew in stature, grew in favour also both with God and man. Society, therefore, has wisely imported this principle into her restraining system; and, by force of it, the woman who has surrendered her virtue, the man who has seduced her into falling, or who has been convicted of malicious falsehood, of fraudulent practices, of breach of trust, of violating good faith, such as opening or betraying a private letter, eaves-dropping, vindictive slander, or any other sin against the so-called code of honour, is tacitly shunned as one who wants nothing of legal criminality, but the courage to defy its penal consequences. And, on the other hand, the man who acts rigorously up to this conventional decency of the world, finds himself so protected by its smiles, that he may cut his neighbour's throat if he will, provided he does it in a gentlemanly way, by tendering his own to a similar process!

And thus by the very restraints imposed upon our passions, ultra their necessary stimulus to provide for the wants of our animal nature, a new impulse to excess is given to us in the gratification of that pride or vanity which, when rationally indulged, supplies the best security for conforming to social usages. Even for this licentious self-complacency, society has found an appropriate and, generally, an efficient remedy in ridicule and laughter; its displeasure may lose its acerbity, but it is not the less painfully felt or the less openly expressed.

This exposition of the working of our passions from their first legitimate use of opportunity to the ultimate abuse of it, may perhaps appear elaborately commonplace; we introduce it to show the infinite gradations that are found in the relaxation of the control of reason, from the first and perhaps momentary indulgence of passion beyond the supply of animal wants, till the chronic indulgence of it carries the offender beyond the limits affixed by law. Strictly and metaphysically, the very first post-prandial glass of wine beyond the allowance which animal want dictates for the restoration of exhausted nature, is a self-indulgence which reason forbids; and is, therefore, as much an act of an irrational animal as self-investiture in a diadem of straw. It disturbs no faculty of ratiocination, it is true; sometimes it improves the power, and if the extra glass is taken with that view, it is a rational, not an irrational act; but if taken merely to please the palate, or to produce a little, brief, pleasurable excitement, the natural appetite being already appeased, it becomes an excess of the restraint which reason imposes, and is, *pro tanto*, the act of an irrational being. So, again, in the trader's case; all trade is, in some sense, a speculation on contingencies; so long as the speculation is governed by knowledge of the market, and by calculations founded on experience, it is rational and legitimate, if the risk is fairly within the limits of his capital; but a single adventure, however small, if not hedged round with these protective circumstances, assumes the gambling character, and proves that the passion of avarice has been carried beyond the limit prescribed by reason; it then becomes the act of an irrational animal. Once more, an honest barber in a borough town may usefully devote a leisure hour to parochial matters in the vestry; he is talkative, fluent, and good-humoured, and, of course, carries all before him; he reduces a rate, or removes a nuisance, and makes himself useful to his neighbours—all this is rational and praiseworthy: elated by success, and presuming on the good will he has secured, he offers himself, at the next vacancy, as a candidate to represent the borough in opposition to Lord John or Sir Robert, the owner of half the town; here reason, for the moment, has ceased to exercise control—vanity has been indulged beyond a useful purpose—he acts irrationally, and is laughed at.

Such casual and trifling disobedience to reason we designate as simple folly. It amounts to no more than a verification of the old adage, "nemo mortalium," &c. Yet, if often repeated, the control of reason is often suspended; if habitually repeated, irrationality becomes habitual; and inasmuch as passion of any kind cannot be habitually indulged without acquiring additional strength by the indulgence, as the cause becomes more powerful the effect becomes more marked; reason is eventually defeated in the struggle, and a state of confirmed lunacy is induced. The approximation to this state may be by degrees almost imperceptible; it may be accelerated by accident, such as wounds, disease, or domestic anxiety; anything tending to unusual excitement may cause more frequent resort to the accustomed irrational gratification; and so it may be retarded by similar accident; the restraint of an unexpected guest, a sudden necessity for travelling or change of residence, even illness of a lowering kind, may suspend the opportunity or the inclination for the wonted indulgence; or the indulgence of one passion may, for a time, be neutralized by the opportunity of yielding to another of antagonistic character. Where casual irrationality slides into chronic irrationality thus slowly and subject to such interruptions, it will cease to excite suspicion that our most experienced men of science, feel and generally avow their inability to define the state; a single drop makes the glass flow over, yet the most accurate eye cannot determine whether the glass will receive one or fifty more without overflowing. It is as difficult to determine the precise moment when passion has overpowered reason and ejected her from her seat, as it often is to fix the minute when death has separated the body and the soul; we feel for the drooping pulse, and put the feather to the lips, and long watch, in silent agony, before we dare close the half-shut eye, and announce that the spirit has departed. A remark in the Report of the Commissioners in Lunacy, of 1847, respecting idiocy, is equally applicable to every class of mania not marked by visible symptoms of organic disease: "It comprises within its limits many intermediate forms, some of which pass into each other by insensible gradations, and are not easily distinguishable by language, although the extremes are well defined and very remote from each other."

It is for this reason that the facts which are commonly presented to medical witnesses, as criteria to test the sanity of a party, are often absurdly equivocal. In the recent inquiry into Mrs. Cumming's case, the attention bestowed on half-a-dozen cats was gravely tendered as a proof of irrationality : as if every old woman in the country had not half-a-dozen pets of one kind or other at her elbow. In other cases, slovenliness of dress, jealousy of female attendants, apprehension of domestic treachery, and even eternal scribbling, have been quoted as evidences of an alienated reason, sufficient to satisfy the physician, whose opinion is to guide a jury. If the question were only whether these were failings inconsistent with a well-regulated mind, that is, a mind governing its will by certain fixed utilitarian principles, such facts would be relevant to the issue; nor can it be denied, that an accumulation of habits decidedly eccentric and motiveless, warrants a suspicion of derangement; though, even in that case, it is only suspicion in aid of proof. No single fact, nor any accumulation of facts, for each of which a possible, though inadequate, reason may be assigned, is, *per se*, conclusive of irrationality : as, for example, had it been proved that Mrs. Cumming was in the habit of walking backwards in the park for half-an-hour daily, what stress would have been laid on such a peculiarity! Yet no man can take a pedestrian tour through Wales, without occasionally witnessing a similar exhibition in well-dressed, sensible-looking young gentlemen; it being well known to all addicted to such amusement, that the intercostal muscles are greatly relieved, especially in ascending hills, by a change to backward walking. Apprehension of domestic treachery is always a favourite topic with the pro-lunacy counsel; yet one of the most eminent artists of the day, whose intellect is as brilliant as his colours, for many years pursued the habit, and perhaps pursues it still, dictated by similar distrust, of baking his own bread, grinding his own flour, and dressing his own dinner, with the same hands that give enchanting animation to his canvas. A single act may be ultra the restraint of reason; even an habitual practice may be motiveless to absurdity, and to that extent, irrational, and yet common sense forbids us to regard it as diagnostic of insanity. It may warrant the conclusion that the agent does not appreciate the force of that conventional code of discipline which we have just described; it may justify censure or ridicule as an error in good sense, a breach of good manners, or an offence to good taste, but it does not argue settled irrationality. Nebuchadnezzar, on being restored to understanding, might have retained in his palace some of the freedoms of his seven years apprenticeship to brutality; he may still have found dress an incumbrance, ablution a painful nuisance, and all the restraints that decency imposes on social intercourse, for a time unnatural. It is probable, from the narrative, that these mementos of his humiliation were not abruptly removed; yet we cannot, consistently with the truth of Scripture, contend that they ought to have been received as evidence of continuing irrationality, for the precise limit of his mental alienation was prophetically fixed; nor would such a diagnosis have been correct, even if he had vindicated the adherence to his bestial habits. He might have plausibly urged, that a sudden change to the warmth of clothing would be prejudicial to his bodily health; that frequent washing was painful to the new cuticle; that the peristaltic action would be impeded by needless control. Such reasoning would at least have been plausible; yet, in modern times, it would have been quoted by Sir Frederick Thesiger as indicative only of the acknowledged cunning of confirmed insanity, and, *malgré* the prophetic limit, a jury would have found him incapable of managing his own affairs, though the Creator had restored his kingdom as well as his understanding.

As acts of irrationality multiply in their frequency or their kind, they may be safely received as indications of a progressive struggle going on between reason and passion, and that the latter is gradually gaining the mastery, but not that the victory is obtained. The abuse of opium furnishes a convenient illustration of our meaning. Its essential medicinal property is so well understood, that men frequently resort to it as a sedative, without duly appreciating it as a stimulant; the dose is repeated till its pleasurable excitement becomes familiar, and then the limit of its medicinal use is transgressed, regardless of its noxious qualities. This is the first act of irrationality. Taken singly, it argues no more than similar excess in the indulgence of wine; it is only the first glass beyond the just supply of natural want: yet a systematic abuse of the drug is a much stronger symptom of the approaching surrender of reason to passion, than a similar abuse of the wine, because the offender cannot be unconscious of the comparative rapidity and greater certainty of the poison; he daily feels that the want and its gratification act reciprocally on each other with fatal effect, not only on his understanding, but on life itself, and yet he courts his enemy and the conflict. But though the symptom is stronger, still it is not conclusive: reason is not yet conquered. The victim himself feels her dictates, and often struggles for a time to obey them. He gradually reduces the indulgence by half-a-grain a day. If he steadily maintains his resolution, reason has triumphed, and he rallies; but in the large majority of cases, resolution fails: he returns to his excess, and then the only question is, whether reason will take her departure before an early death effects her total separation from the body whose passions have estranged her.

So, too, where the acts of irrationality multiply in kind, as well as frequency or degree. If the same excess of vanity that leads an honest barber to propose himself for parliament, tempts him to array his person in military uniform, and decorate his breast with spurious clasps and medals, we cease to ridicule his folly, because we begin to doubt his sanity; it is a step in advance, but it is not conclusive. We remember an instance of this kind during the short peace of 1814. A youth of eighteen, the follower of a very humble and peaceful occupation, was not only accustomed to assume the warlike garb, but more than once thrust himself, in his borrowed plumes, into the gayest military circles. He was soon detected, and punished with deserved ridicule: yet he was not irrational; and on the contrary, for eight-and-thirty years he has maintained a high reputation for accomplished vice, without the good fortune to excite a transient suspicion of any intellectual deficiency! To proceed with our illustration; let the barber, in addition to his other antics, offer his hand to Miss Burdett Coutts, or tender his acceptance for a few thousands for discount at the Bank of England, his preposterous pretensions tend largely to the same conclusion, though still they do not establish it; for marriage with a wealthy heiress, or even credit to a large amount, may enable him to buy a seat in parliament, or establish himself as colonel of a yeomanry regiment, and thus realize his dreams. All these supposed extravagancies are but so many cumulative proofs of the excess to which vanity is carried beyond its use as a utilitarian principle; they may terminate in alienation of mind, but do not prove her actual departure. If, however, simultaneously with these absurdities, the unhappy wretch now and then mistakes a grate of hot coals for his chair, or seeks to draw a glass of ale from the spout of a boiling kettle, or shaves a customer's head in lieu of his chin, this multiplication of irrational acts, in kind as well as degree, justifies the conclusion that reason has actually vacated her throne, though the precise moment of the abdication may remain as problematical as ever.

There is a remarkable feature in that perpetual struggle between passion and reason, which terminates in insanity in the manner we have described. From the first commencement of the conflict to its termination, reason is forewarned of the ultimate result. A single glass taken in excess, or a cheerful glass, as it is frequently called, is always followed by some proportionate depression when the power of the stimulus is exhausted. If the abuse has been but slight, the depression is transient, and speedily removed by the excitement of business and daily duty; if the abuse has been considerable, the depression will cause a temporary incapacity for duty; if it has been unbounded, physical incapacity supervenes, and this unconsciousness is followed by utter prostration of spirit. These stages of intoxication are well understood by the vulgar phrases of "fresh," "drunk," "gloriously drunk." The excitement is well observed, and tersely described, by the class in which it is common, but the subsequent depression eludes their observation. The opium-eater exhibits this alternation of gaiety and sadness in a more decided form. In his case, intoxication is elysium, and its sequence, hell: and so it is, more or less, with every struggle between passion and reason. The calmness of self-possession gradually becomes unknown. We perceive this feature clearly in the familiar instance of intoxication. It is equally marked, though less distinguishable by the unphilosophic eye, in all cases of contention between the appetites and the reason. Gratification gives delight, but it is transient, and vanishes in self-disgust, till new gratification revives the delight in a less intense degree, and for a still more transitory existence: eventually, even gratification itself palls upon the taste; all pleasure is lost, and incurable despondency ensues. Miserly avarice, perhaps, is an exception to the rule; but if an exception, it is only because the passion is, from its nature, insatiable, and absolute gratification unattainable; and even avarice to be an exception, must be miserly, for when it assumes the form of gambling, the opium-eater's languor is bliss, compared to the gamester's remorse.

It cannot be doubted that these retributory warnings, inseparable as they are from all excess in the indulgence of our animal passions, are mercifully designed to give reason time to rally; to afford fair opportunity for reflection; to enable her to resist the next temptation with more fortitude and effect; for it is indisputable that up to a certain point, when physical suffering has actually exhausted the energy of the mind, its power is never so great as when the mere animal is subdued into torpidity by satiety, the excitement of sensual gratification having worn itself out: in religious language, conscience then begins to awake; in metaphysical language, we should say that reason then exerts her power; she looks back, she calculates, she estimates the past and plans for the future; and she resolves. We well know that by her own strength alone, her resolutions will be wanting in constancy; but we are considering the matter as philosophers, not as divines, and we therefore abjure discussion of her self-sufficiency: we only aver the fact that reason is most awakened and most powerful in the intervals of animal excitement, and it is a most important fact in that psychological theory for which we are contending: it is the remark of every commentator that our Saviour, when tempted in the wilderness, an occasion when he stood alone in his humanity, found himself in a state of almost super-human endurance; that the design (if it is permitted so to speak of the mysteries of revelation) was to add all possible force to the temptation by the predominance of animal want; it was expedient to show to us for whom the atonement was made, that the sacrifice was immaculate; the very nature of the temptations offered, and of the indignant repulse given to them, proves that his humanity was, as it were, momentarily deserted by his divinity; he repelled Satan by reference to God, and not by any inherent power in himself: and to make his human innocence, if we may be allowed the expression, more conspicuous, the trial was aggravated by physical suffering of the precise kind that the temptation appeared calculated to relieve. We are entitled to infer from this that reason is the weakest, when passion or desire is at its culminating point; and that as desire is satisfied, reason resumes her sway.

And it is through this interlocutory cessation of strife that, in the large majority of cases, in the earlier stages of the conflict, reason recovers her superiority once and for ever: shame at the self-exposure (for reason always desires to veil her own infirmities as well as those of the frail tenement she inhabits), apprehension of more serious consequences, and where principle has been instilled by education, a consciousness of sin, combine to strengthen determination for the future; and temptation resisted with success, loses power after every defeat.

Nor is it unfrequently that we find, in more advanced stages, that a counteracting influence is brought in aid of reason, when beginning to faint from the exhaustion of reiterated assault, by another inevitable result of habitual self-indulgence; the sickness and debility of the organs that it has sought to gratify. It needs not the authority of science to assure us, that in whatever direction passion is indulged to excess, actual disease of the organ thereby kept in constant excitement, will sooner or later follow; and from the sympathy that exists between every portion of the body and the brain, and more especially and perceptibly, between the stomach and the brain, affections of the nervous system are usually the first visible fruits of organic derangement. It is not necessarily the case that the cerebral substance is appreciably altered in its structure: when that occurs in certain parts of the brain, the symptoms of insanity are no longer equivocal; but long before disease has attained that height, the patient is conscious of pain, dulness of perception, impairment of memory and irregularity of thought: in some cases slight epileptic or paralytic affections add their premonitory hints: and in all cases the taste for the favourite indulgence is temporarily checked. The alarm thus given, and yet more, the abridgment of opportunity by the physician, and the languor of the depraved inclination, combine to give another respite to reason. A singular instance of this once fell under our observation: a young clergyman, from domestic trials of a very severe nature, and not the less severe because induced by his own misconduct, betook himself to drinking, as well as other indulgences of a yet more debasing character; being the incumbent of a remote country village, he was able to continue his profligate course for a few years without attracting the notice of his diocesan. At length his nervous system became so shattered that exposure was inevitable; compassion for his state saved him from degradation, but he was suspended for two years; this ignominious sentence compelled his return to the parental roof, where for many months his life was despaired of, though reason never absolutely forsook him; sickness, however, accomplished her end. There is still too much ground to fear that the reform is based on no higher principle than prudence; however that may be, his moral conduct has ever since been correct, and all his intemperate habits abandoned; this case was singular, because he was at once pronounced insane by the concurrent opinion of his medical attendants, first in the country, and then in London, while maternal affection denied it; with natural jealousy she repudiated all the treatment recommended under that impression, except so far as to deprive him of all opportunity of self-indulgence. Nature and reflection, aided by bodily suffering, did the rest.

The conclusion at which we arrive from this theory of mental pathology is, that the sense of responsibility, though gradually decreasing at every successive stage, is never wholly lost till the alienation of reason is unequivocal: calculation of consequences not only may exist in a man whose passions have uninterrupted sway for three or four days in every week, but nature has provided intervals for reflection and calculation arising from the very cause of the disease, and has given to the mind during such intervals, a peculiar adaptation of tone to avail itself of the opportunity thus afforded for weighing consequences.

It will of course be at once understood, that our theory is confined to those cases in which there is no possibility of forming a diagnosis from antecedent circumstances, or physical development. If a man has been subject to epileptic attacks of aggravated character, it may be safe to predicate insanity, from eccentricities and absurdities of thought and action, which otherwise would only indicate and amount to folly. If there is reason to apprehend an hereditary predisposition, and the head, the complexion, or other features, indicate a scrofulous habit, the same latitude of judgment must be allowed. The state of the eyes is often symptomatic of cerebral pressure; the expression of the features, well understood though difficult to describe, may disclose aberration of mind at a glance; the idiotic vacancy, though it may be casually assumed by actors like Liston, is, when permanent, a symptom too decisive for mistake: in a word, wherever there is clearly a predisposing cause from physical injury to the head, constitutional affection, or visible organic defect, it is needless to go very minutely into evidence of conduct. The cases of perplexity are those in which peculiarity of conduct alone, and wholly unattended by decisive physical symptoms, affords the evidence by which disease of mind is to be determined; such cases are considered to resolve themselves into metaphysical subtleties; medicine, as a science, being supposed to have little to do with them. The action of the mind upon the body is almost as great, though not so apparent as the action of the body on the mind; hence, by constant observation of the characteristic symptoms of those labouring under undoubted mania, the physician may infer the existence of incipient mania from similar phenomena in a suspected party; and to this extent his experience is entitled to weight; but where all such phenomena are wanting, or are uncertain in their appearance, some other theory, it is said, than that of physical disease must be suggested to account for derangement of the mind: we have on a former occasion expressed a doubt whether in any case mental derangement is ever found unattended by some altered state of nervous matter; we adhere to that opinion, and the theory we have ventured to enunciate, is in unison with it. But the organic disease may be too obscurely developed to guide the judgment; or its appreciable symptoms may consist with other affections notoriously unconnected with mania, or insufficient to account for it. Restlessness, indigestion, increased arterial action, and many other irregularities of the system, are found as often in sane as in insane patients; after mania has become confirmed, and especially in those cases where it is incurable, the bodily symptoms assume a common type, varying a little according to constitutional habit or the violent or melancholy character of the insanity; when the mind has clearly taken its final leave of the body, as regards its proper control over it, the animal nature, thus left to itself, is, though animated, essentially a passive substance; that, moulded by the same hands and sustained by the same nourishment, and governed by the same principles, will assimilate itself to any other substance of the like nature, so far as it is exempt from any peculiarity of disease or organization. In such cases we may expect to find a general uniformity of symptom. Where, on the contrary, the alienation of mind is not irremediable, its morbid action on the body will be imperfectly developed; and though local disease may exist, the actual seat of it may not be discoverable by any symptoms peculiar to itself. In such cases we are compelled to resort to some pathology of the mind to guide our diagnosis.

It is not, however, for scientific purposes that we have thus suggested a principle on which such mental pathology may be based; our idiocrasy is a subject of study for the practical statesman as well as the physician; all peculiarity of temperament, and the causes which elicit it, are well worthy of consideration in the dynamics of legislation; it seems strictly within our province to aid in supplying the elements of legislative calculation, and this is our apology for pursuing the inquiry into fields where science rarely trespasses.

If our theory is correct, it affords a clue to the solution of the problem that has long perplexed the most acute among our lawyers, as well as the most learned among our medical professors. "Where shall the limit of responsibility be fixed?" The *mens capax doli* is, as everybody knows, the criterion of lawyers; but, except in the case of children, they always have recourse to physicians to interpret this indefinite standard. A few years since peers and judges met in solemn conclave to evolve out of the confusion of ethics and metaphysics, in which both professions had become inextricably plunged, some term of more definite meaning. The united wisdom of their lordships broke down, as seems to be the inevitable lot of collective sagacity in modern times. It was announced by their supreme authority that a capability of distinguishing right from wrong should henceforward be the measure of responsibility. This was not even a step in advance; it only substituted for one expression of doubtful meaning another still more unintelligible. As we long since argued, "right and wrong" are arbitrary terms, and no two people are exactly agreed in their application of them to any given deed of humanity. The only practical result of this learned attempt to define that which is from its nature undefinable, has been to give sanction to a judicial usurpation of the functions of a jury; and to a certain extent this has worked well, for our judges are far less credulous of insanity than our juries, and so are we in respect of its apology for crime. It is, however, still found that in all cases where medical opinion is required, the "right from wrong" craniometer is unsatisfactory to our professional brethren, and not always conclusive with a jury, notwithstanding their wonted deference to the court; the problem of responsibility, therefore, still remains unsolved.

Bearing constantly in mind that the problem never arises in cases of unequivocal insanity, the difficulty may be stated thus: We find a man apparently in good bodily health charged with a breach of our criminal code; the offender has been long noted for eccentricity, and the crime appears to have been committed without obvious motive: is such a man to be held responsible like other men?

The corollary from our theory is, that criminality, moral or legal, and, as regards the argument, it matters not which, is not only consistent with the progressive alienation of reason, but is at once the cause, and the invariable precursor of its final departure, excepting only such cases as may be explained by physical indicia of a determinate character. All indulgence of our animal propensities beyond the limit that is necessary for the support and propagation of animal nature, is, morally or religiously speaking, a crime; that is to say, it is a transgression of the boundary which the law of God has appointed to the gratification of our animal appetites. The law of man has been less severe in placing the boundary; its restraint only begins when self-indulgence becomes injurious to the reasonable gratification of others; the former code has for its object to fix our responsibility to our Creator; the latter code to fix our responsibility to society; but the subject-matter of either code is equally the gratification of our appetites, and the object of both is self-restraint; conscience gives stringency to the first, and punishment to the last, while reason is the guide to submission in both cases; disobedience to our guide is visited with immediate penalty in the one case, and with future penalty in the other; and inasmuch as immediate punishment is always more potent as a check than remote punishment, we find a far larger proportion of mankind acting in disobedience to reason in reference to the law of God than in reference to the law of man; hundreds and thousands daily indulge in many a glass too much for actual necessity, who would be horrified at the idea of being picked up in the street in unconscious drunkenness; yet the offence is the same except in circumstance. The incident of publicity brings it, in the one case, within the category of municipal crime, but reason is as much offended in the one case as the other; her restraint is despised in both instances alike; criminality instantly attaches, but responsibility is instant or remote, according to the code which has been violated; in the first stages of criminality, consequences are calculated with accuracy and even anxiety; as it becomes more frequent, frequent impunity becomes an element in the calculation, till reiterated experience of impunity bids defiance to all calculation, and the offender persists in his career, regardless of consequences. This is the precise epoch from which common observers are apt to date the moral symptoms of mental aberration; nor can this excite surprise, for the *debilitation* of reason by reiterated defeat in her conflicts with passion, is a theory that has never been propounded: that conscience becomes callous by resistance, is a doctrine enforced hebdomadally; but conscience is a faculty so distinct from uneducated reason, that it often becomes obliterated before reason has attained its maturity.

Disregard of consequences does not necessarily imply inability to calculate them. A man who cannot swim may plunge into the sea to save a child from drowning; in his generous heroism he disregards consequences; he is perfectly able to calculate them, and may have argued the folly of such self-sacrifice only five minutes previously; but generosity is a passion, though, unfortunately, of rare occurrence; he yields to the impulse of passion, and defies consequences; for the moment, reason has lost her influence; and if he fails in his object, but is himself saved, he will probably assent to the selfish comment, that he was a fool for his pains; yet, in such a case, or for such a cause, who would venture to denounce him mad; charity herself could not deny his responsibility, though she would plead the generous feeling as a fair ground of exemption from punishment. The argument will hold good in the case of the baser passions, as well as in the noblest; it is only the palliation that fails.

Our corollary is also sustained by the strict analogy which is observable between the progress of crime and the progress of mental alienation; the sophists of antiquity were as familiar as ourselves with the graduality of degeneration; the chaplain of every gaol listens daily to confessions that prove the apothegm "*nemo repente turpissimus.*" The first watch abstracted from its owner ticks punishment into the ear of the thief for hours; the watch goes down, and apprehension goes down with it; some "fence" buys it for a sovereign, and the delinquent finds himself in wealth for four-and-twenty hours; such, however, has been his alarm after the first offence, that reason resumes a temporary sway; he reckons up the risk and resolves better things; but, meanwhile, he starves, and starvation is not the less painful in the recollection of his recent day of plenty and debauch; he will try the adventure once more —only once more; if he succeeds he will husband the resources it supplies, and look out for honest employment; it is reasonable to allow himself a better chance; if he can "twig" a purse, his profit will be greater and give him more time to seek for occupation; he watches an unsuspecting victim receiving dividends, and aiding audacity by ingenuity, again succeeds—twenty sovereigns reward his second crime; he reckons with more confidence on impunity—he finds his reckoning right; a fortnight of idleness and profligacy repays him, and crime now becomes his trade. Planned robberies, well "got up," succeed to petty thefts, and these, in turn, are superseded by higher and more profitable crime; the wants of nature are well supplied, and passion, beyond her wants, is abundantly indulged; indulgence adds craving to the appetite, and appetite must be satisfied, reckless of consequence. This is the ladder by which the highwayman and murderer ascends the scaffold.

It matters not what may be the character of the crime; it may be arson, it may be rape; the first successful gratification of vindictive feeling leads by similar progression to the one; the first flirtation of simple sensuality, unchecked, if not encouraged, leads by the like gradation to the other; in all cases progress from venial to bad, from bad to worse, and thence to extremes, is the invariable trait of a criminal career; consequences are first calculated with anxiety, then merely weighed against immediate gain, and, finally, disregarded altogether.

Here we find a perfect identity of character with that form of mental alienation which is (apparently) distinct from organic disease. There is no abrupt transition, no sudden metamorphosis, no marked convulsion of the system, no violent disturbance of accustomed habit; cause produces effect by obvious and natural process. Each successive step in either progress is characterized by the same traits. The first is so slight an interruption to the daily path that it is taken almost unawares; then conscience, the barometer of morals, indicates a fall to a lower level; consequences are now calculated with alarm that magnifies their danger, and the calculation always arrests, and sometimes prevents, further descent. Passion at length revives with aggravated strength, and suggests that reason has overrated risk. Then comes the second step, again followed by self-reproach, but with pangs less durable, and apprehensions less lively and defined. Thus the interval is reduced between the second and third, and that reduction proceeds in geometrical progression; then step follows step with a rapidity that admits of no check, till descent is terminated by the bottom of the abyss. In both cases the gravitation is occasionally interrupted; opportunity is adeemed by change of circumstances, waning energy or lowering sickness: these present a temporary obstacle, like a projecting crag that breaks the precipice, and extend a momentary reprieve; but though strength may be recruited, it rarely avails to re-ascend the heights; the downward tendency has become habitual; even the sensation of reckless descent has acquired a charm; desperation itself is not without a compensating power, and the temporary self-possession succumbs to it.

Identity of object is as marked as identity of progress; the object in both cases is self-gratification, or, more correctly speaking, gratification of passion. And here the legal criminal is often less culpable than the moral criminal, and therefore more entitled to the protection of irresponsibility. Our passions being designed for the support and perpetuation of our animal nature, concession to them, up to a certain point, is, as we have before observed, legitimate; but the pauper can with difficulty provide gratification even up to this legitimate extent:

when his superiors complain of hunger he complains of famine; they talk of fashion and overheated rooms, while he bewails both cold and nakedness. If both transgress in availing themselves of the opportunity for excess, why should the rich man have a better claim than the other to the privilege of irresponsibility? We admit, however, that both are offenders, whatever may be their comparative temptation; for the gratification of passion, ultra the demands of animal nature, is their common object. The pickpocket has no abstract love of stealing for its own sake, unless here and there vanity may prompt him to exhibit his excellence in art. As a general rule, he steals to get a dinner; and he steals in preference to working, because the labour is less and the profit greater: he provides a dozen meals in less time than the honest navigator can earn one. Thus the animal love of rest is gratified simultaneously with the desire for food; he has enough for the hour and to spare; destitute of other resources for amusement, he feeds his passions with the surplus; and steals again to satisfy the cravings of stimulated appetite, though at first he only stole to appease the same appetite in its natural state. The object of the moral offender is precisely the same; he, too, by the temptation of opportunity, has stimulated passion to a pitch of morbid craving, and, *coute qu'il coute*, it must be satisfied. He need not steal, but he opens a second bottle, and were it not in his cellar, he would steal rather than want it: not perhaps on the first occasion, nor yet the second, nor indeed for many. At first he pays, then pays on credit, then borrows; and when means and credit are exhausted, he defrauds or steals, and descends to the class of legal crime; for gratification he must and will enjoy, beyond the mere wants of animal necessity. If his means are too ample to exhaust, the object is still the same—the amplitude or the insufficiency of means is a mere accident in a philosophic view. We have taken but one, and that the most familiar subject of inordinate self-indulgence. We might pursue the analogy through all the range of human passion—lust, anger, revenge, jealousy, envy, avarice, pride, vanity, ambition, are uniform in their action whatever may be the social position of the man. Crabbe, a name scarcely known to the present generation, though venerated by their fathers, has beautifully illustrated this truth in his village tales. The only essential difference in the positions of the legal and the moral criminal, is, that the self-indulgence of the one is dangerous to the community, and of the other only to himself; in the former it is practised at the public expense, and in the latter at his own.

But this is a difference that points to a plausible objection to our theory. "How does it happen," it may be urged, "that the large majority of legal criminals are of an age so young that it would be absurd to contend for the triumph of passion over reason? Their reason is not matured; they are, for the most part, too ignorant to appreciate, or even feel, her restraint. Who ever heard of a boy of the age of fifteen setting up the defence of insanity to a charge of pilfering?"

The difference we have mentioned affords the answer. If the child of fifteen, or five years younger, has displayed art in avoiding detection, it is conclusive that his reason, however limited, has sufficed to tell him that he has broken the law. He has disobeyed the dictates of reason no less than the adult. It is conceded however, in his case, that reason cannot have been extinguished altogether by perennial defeat in his struggles with passion. Even passion itself, at so youthful an age, rarely attains its strength; but legal criminality being dangerous to the community, it is necessary that a system of prevention and detection should be instituted, and as juvenile crime is less artificial, it is more easily detected, and thus the young criminal is arrested in his career long before the triumph of passion over reason is achieved. Our theory is, that every self-indulgence, beyond the claims of animal want in its natural state, is opposed to reason; and, as an act uncontrolled by reason is so far an act of insanity, in any strict and philosophical sense of the term; but we do not, therefore, say that reason is unseated; her actual expulsion from the animal man, is only effected by the constant repetition of irrational acts at shorter intervals and in greater variety, so that the contempt of reason becomes chronic and habitual.

If the case of juvenile depravity is followed up, it will be found to sustain our theory in a remarkable manner. At every Middlesex Sessions, the judge, Mr. Serjeant Adams, complains in strong language and with just indignation, of the reiterated appearance before him of the same children; punishment has no reforming power; boys of ten and twelve are again and again committed, imprisoned, and flogged, and then discharged only to re-appear in court within a month to receive the same sentence. In all such cases we are driven to a sad alternative—either the animal wants of nature cannot be legitimately supplied without offence, an explanation too frequently too true; or, though they can be legitimately supplied, reason has wholly lost her power to restrain illegitimate excess. If this branch of the dilemma is adopted, then by our theory we do arrive at the conclusion that reason has become extinct by indulgence, even before she has attained maturity!

Thus far we have shown that the progress and the object of crime in its legal sense, and of the estrangement of reason, are identified. We will pursue the parallel to the final results, and there we shall also find that the same similarity obtains.

The late Lord Nugent, a man very dear to those who knew him well, was distinguished as the champion of the opponents of capital punishment. Agreeing with him generally in principle, and much associated with him in most of his benevolent undertakings in his county, he was anxious to enlist us in this crusade; but there was one difficulty in the case which even his lordship, ingenious and dexterous as he was in parrying all objections to mounting his hobby, confessed his inability to remove. If you abolish capital punishment at home, how can you retain it in a penal colony? The principle on which you contend for its abolition is, if just, a paramount principle; that life being the most valuable gift of God, as affording while it lasts an opportunity for reform and repentance, man may not abridge it, and thus deprive the sinner of his eternal hope. If the legislature adopts this principle, then it must extend to every place within her jurisdiction. But in a penal colony, secondary punishment is exhausted, and expended, too, without reform: then what remains but the extreme penalty? Suppose a man resolutely determined on suicide, and he is self-placed beyond the pale of law; he may sally into the streets and plunge his knife into every one he meets with impunity. When taken, they can but hang him, and he is resolved to die already. He has the prussic acid in his pocket; before he has reached the station he will be a corpse. Who can deny that, were a pre-knowledge of the man's status possible, it would be right to kill him at once, on the same principle that you kill a rabid dog? The contumacious criminals of a penal colony are in a position precisely similar, when this contumacy proves that secondary punishment is fruitlessly exhausted. Self-placed beyond the pale of law by offending beyond the possibility of further punishment, they become dangerous animals, whose extinction is indispensable to the safety of their fellow-convicts.

But it is not as an argument against this extravagance of modern humanity that we quote it here: it is a convenient illustration of the status to which man is reduced by crime. Absolutely unfettered and unrestrained, because the power of punishment is gone by exhaustion, what is he but a rabid animal; and doubly dangerous because, though reason has departed, cunning remains. We are not speaking of the convict criminal, but of the incorrigible criminal among convicts. Taking the convicts as a class, many among them show, by their subsequent conduct, that reason recovers her sway when the opportunity of self-indulgence is long suspended. A compulsory self-denial is wrought into a habit, and they again become industrious, prosperous, and orderly members of the social body. These, however, are exceptions; and it is remarkable that these exceptions (we speak from information given by a gentleman who, very undeservedly, spent five years among them, when at length proof of his unjust conviction obtained his full pardon,) are always to be found among the educated portion of them; but the bulk settle down into a state scarcely removed from bestial irrationality. A striking instance of this utter degradation is to be found on parliamentary record. Six convicts escaped into the bush, without food or the means of obtaining it, except a single axe. It served to provide them with the food of cannibals, but no other. They successively fell under the axe to provide a horrid meal for the survivors, the last of whom returned to Hobart Town to confess the dreadful tragedy, and be hanged! In other cases, the miserable beings would draw lots who should die; the victim was promptly accused of some capital offence in contemplation, and the false accusers received the usual reward for discovery; the price of blood being expended by the others in clandestine purchases of spirits for a night's debauchery! Practices which no pages may record, except the annals of our criminal courts, were at one time all but universal. The disgusting horrors of convict life, as exposed on a parliamentary inquiry some years since, admit of no parallel in the history of man, except what might be found in some of our lunatic asylums at the beginning of the present century. It is, alas! too true. The lunatic asylum and the penal settlement once stood unrivalled, except by each other, in all that is dreadful and disgusting; and for a very sufficient cause—man in the possession of his physical power, and deserted by his reason, is at once the most profligate and the most dangerous animal in God's creation.

We may find another example of similarity of result in our workhouses at home. Their inmates, in the class of casual paupers, usually include many who have also been inmates of the gaol. In our prisons the discipline is necessarily severe, and there is a sufficient staff to maintain it, and sufficient power to enforce it. It is not so in the workhouse; and hence, while we rarely hear of disturbances in prison, we constantly read in the police reports of the most violent and motiveless outbreaks in these pauper asylums. The common apology of the offenders is, that it has all been "by way of a lark." After making all possible allowance for the romping turbulence of half-a-hundred young men and women thrown unexpectedly together, it is difficult to account for the absurd and causeless violence exhibited on these occasions, except on the theory of transient irrationality. Boys, on the breaking-up for the holidays, used, in our young days, to break up forms, and desks, and school-room furniture, with a ruthless hand; but it was done as the symbol of emancipation from scholastic rule, and of a return to the freedom of home. The emblem might be rude, but it had its meaning. It is difficult, however, to find any emblematic expression in the wanton destruction of property that generally attends the "larking" of our casual paupers; but if we ascribe it, as we may fairly do, to a spirit of mischief and ill-nature, we are justified in classing it with those other eccentric irregularities which, on our theory, are acts of irrationality, though too transient to imply more than a progressive step towards total alienation of mind.

This combination to effect a common purpose of turbulent mischief, exactly corresponds with the disposition of confirmed lunatics of the violent class, should accident bring them together unwatched by their attendants; the only difference being, that they will, perhaps, injure each other as much as the property within their reach, so that the concert or combination is less conspicuously developed. This may argue a nearer approach to total alienation of reason; but, nevertheless, mischief is the type of irrationality in both cases.

Another resemblance in the results of acknowledged irrationality and incorrigible criminality, is to be found in the absence of common decency, as regards the infirmities of nature, or social decorum. There are occasionally, though rarely, positions in life where reason herself prescribes a temporary departure from that modesty which is a part of our fallen nature, and was the first evidence of our fall; but when no emergency arises, this insensibility to appearances is one of the most certain signs of that degradation which attends the departure of reason. The same feature is broadly developed not only in the worst classes of our convicts, but also among our disorderly paupers. Duty has sometimes called us to the houses occupied by the most degraded of our metropolitan poor, and there we have witnessed scenes that argued a total deprivation of moral sense; but we have invariably found, even among the poorest, that where character remained unsullied, the decencies of life were, at least outwardly, observed.

It would not be difficult to trace the close similarity of results in further points. The same jealousy and distrust, the same submissiveness to authority in constant and severe exercise, accompanied by the same vigilance of cunning to escape from it, the same habits of dissembling and deceit, the same restlessness of body and anxiety about trifles, and more than all, perhaps, the same indifference to danger, strangely attended by awe of corporal punishment, mark both the criminal and the irrational being. It is a curious inquiry, but our limits forbid us to prosecute it further. We have, we think, shown that in progress, in objects, and in results, there is an identity of character in crime and insanity. This naturally leads us to investigation of the cause; and we have suggested a theory which gives a common origin to both, and warrants the conclusion that, however they may differ in name and in responsibility, insanity and criminality intend the same status of the human being in regard to his rational or intellectual functions, excepting only in those cases where a morbid derangement of the organic structure is apparent.

We anticipate an inquiry which will be made to test our theory. Does it appear that insanity, in its perfect and incurable form, is found more frequently in the convict class than in others? We have no accessible statistics to supply an answer to this question; nor should we be satisfied with that answer, even if it were favourable to our theory. Scarcely a week passes over but we find men of undoubted science and experience, with ample opportunity for observation, and abundant learning to guide it, who, nevertheless, differ widely in their opinion in any given case. We ascribe this diversity of opinion to the want of any acknowledged principle of analysis; but if, with all these advantages to aid them, our most eminent authorities are so much at variance, what reliance can be placed on the report, however faithful in intention, of some two or three young and comparatively unknown men, successively sent out in charge of a convict ship, or a convict station? But if the answer were all worthy of confidence, and adverse to our theory, it would still be inconclusive. The great majority of convicts are young and in the prime of life. From the hour of their conviction they are placed on the strictest system of restraint and dietetic discipline; they are debarred from all opportunity of excess; even their tempers are kept in constant check, and their bodily health is sedulously watched. Under such very favourable circumstances, all morbid action is likely to be arrested, and reason may often be restored. Nor is it part of our case that the convict, as such, is necessarily advanced to that stage of progression when reason becomes prostrated by defeat; though, in the case of the contumacious convict, self-placed beyond the power of secondary punishment by reiterated and unceasing crime, we believe the consummation to be complete. Our theory, therefore, remains intact by the result of the supposed inquiry, whatever it may be; but the inquiry is one of such importance, that we trust the legislature will require such returns as the nature of the case allows of being made.

A similar test may be suggested in the case of the Society of Friends; for, more accustomed as they are by education to habitual self-control, insanity, according to our theory, ought, among them, to be of comparatively rare occurrence.

Here, too, as we believe, the statistics are not obtainable with sufficient accuracy to justify absolute reliance upon them; but so far as we have them, they are most favourable to our views; we may observe that these statistics are deficient in one important feature, a defect which they share in common with all lunacy returns; no distinction is made between patients labouring under an insanity induced by predisposing causes of acknowledged influence and those cases in which a predisposing cause cannot be detected. It is to the latter alone, as we have r peatedly said, that we propose to apply our theory. It may also be observed that hereditary taint, where it exists, is more likely to be propagated in the Society of Friends, because its members notoriously intermarry with each other, with a frequency not occurring in any other limited circle; and lastly, it should be noticed that, from the peculiar constitution of the society, it is their practice to send to their asylums very slight and equivocal cases of derangement; these swell the number of the inmates, though it may be doubted if they are of a type sufficiently decided to affect any theory founded upon such statistics. Subject to these remarks, we may quote the following calculations of Dr. Thurnam. The average number of the Society in England and Wales during the twenty years, 1820 to 1840, appears to have been 17,900 of all ages: for reasons which he does not explain, but which we may presume to be, that insanity does not usually occur in extreme youth or age, he takes only 10,000 for the basis of his calculation; he assumes that the Retreat at York embraces in its experience all cases that occur in the Society; this experience will give 1 in 2196, for the proportion of original (as opposed to *recurring*) cases of lunacy in a population of 10,000, annually: but he adds to the cases of the Retreat such a number as he considers may not be sent there, being left to private treatment at home; and thus corrected, he estimates the proportion at 1 in 1790. By including the experience of the Retreat at Bloomfield, near Dublin, he further increases the proportion to 1 in 1590, and there seems no reason to doubt the general accuracy of this result, though it is not very clear whether he means to limit this larger result to Ireland, or extend it to England.

Much variety of opinion has prevailed as to the comparative exemption of the Society of Friends from this calamity, in relation to other classes of the community. Dr. Thurnam, in quoting the conflicting opinions of Dr. Burrows, Dr. Jacobi, Dr. Julien, and Dr. Haslam, inclines to the side of partial but not signal exemption; so at least we construe his remarks, though they are avowedly made with hesitation: he states distinctly, however, that the statistics of the Retreat "show that intemperance and other causes of frequent operation in the world at large are rarely met with as causes of insanity in this community;" the chief ground of his hesitation in arriving at a more definite conclusion, is the absence of data on either side of sufficient accuracy. Let us, however, compare his results with such data as we possess. In the Report of the Commissioners in Lunacy of 1844, a table is given, extending only to the pauper class, in which the proportion of cases to the population of England and Wales is given as 1 in 1019 for 1842, and 1 in 980 for 1843; and in the same report, a "General Statement" is published, "of the total number of persons ascertained to be insane in England and Wales," under date of the 1st January, 1844; this number is 20,893, which, compared with the population according to the census of 1841, will give 1 in 784 for the index of English and Welsh insanity; but this includes only such patients as are inmates of public institutions, or found lunatic by legal inquisition: a large correction must be made for the unascertainable proportion of private patients, in families whose circumstances do not compel them to transfer a deranged relative to a public asylum; if we estimate this as an addition of a twentieth part* to the sum of lunacy stated by the commissioners, and such an estimate seems moderate, we obtain 1 in 745 for the ratio of lunatics to the population, according to the census of 1841.

According to Dr. Thurnam's experience, predisposing causes are to be found equally among the Society of Friends as among other bodies, with the single exception of intemperance: the term, of course, not being confined to its vulgar acceptation of excess in drinking, but including every form of excessive self-indulgence. If, with this exception, the predisposing causes operate with equal force in the Society of Friends as elsewhere, it follows that any greater prevalence of insanity in other classes is ascribable to "intemperance;" according to the calculations we have just given this greater prevalence is measured by the difference between 1 in 745 and 1 in 1590, or very nearly one half: thus, so far as the data that we do possess extend, one half of our cases of insanity are proved to be caused by excess of self-indulgence, or, in other words, by the habitual gratification of our passions, ultra the necessary wants of our animal nature.

If we were to found our calculations on a more recent report of the commissioners, the case would be yet stronger; for, on the 1st January, 1847, the number of lunatics had increased to 26,516, and this would give the ratio of 1 to 618 as the proportion of lunatics to the population, instead of 1 to 715: so that by the same process of reasoning, considerably more than one half would be shown to be the victims of habitual self-indulgence; this, however, would be fallacious, because the population of the country in 1847, largely exceeded the census of 1841; we need not resort to any fallacy to strengthen the argument, for the statistics contained in the report of 1847 disclose another fact which

* In the Report of 1847, the commissioners observe that the higher and middle classes contribute their share of the lunatics of the kingdom in the proportion of 5000 to 18,800; this would justify the addition of a much larger part than a twentieth, it being among those classes that private patients are usually found. We refer to the fact principally, however, for another reason: it justifies an argument subsequently adopted in the text in favour of the identity of lunacy, induced by habitual intemperance, with that status of hardened criminality which otherwise would appear to be almost confined to the pauper class.

will far extend the proportion which we have assigned to self-indulgence as a predisposing cause. It is stated (p. 274) that, "of the entire number of lunatics in workhouses, computed at 6020, or thereabouts, two-thirds at the least, or upwards of 4000, would be properly placed in the first class," that is, in the class of "the weak minded, imbecile, or idiotic." If this analysis of so large a proportion as one-fourth of the lunacy of the country, is accurate, it follows that two-thirds of the whole body of 26,516 fall within the same class, leaving only 8840 whose insanity is to be accounted for by extraordinary or predisposing causes.

We have stated that the difference between 1 in 745 and 1 in 1590 is the measure of insanity caused by intemperance; but if we are to infer from the quotation just made from the report of 1847, that two-thirds of the sum total of insanity consist of imbecile or idiotic cases, we must deduct two-thirds from 745, and then the measure of insanity caused by intemperance will be the difference between 1 in 249 and 1 in 1590: or, in other words, more than five-sixths of the insanity in England and Wales is to be ascribed to habitual self-indulgence.

Our argument, then, is briefly this:—

In the Society of Friends, where intemperance is not a predisposing cause, one person in 1590 is insane.

In society at large, where intemperance is added to other predisposing causes common to both, one person in 745 is insane.

Of these 745, two-thirds are imbecile or idiotic, being a form of insanity almost universally traceable to organic malformation or to senility: deducting two-thirds, 249 will remain for the number whose lunacy is to be attributed to causes of which the operation is more or less problematical, intemperance being one of them.

If then only one in 1590 is insane, where intemperance does not operate, and one in 249 where intemperance does operate, other predisposing causes being common to both, it follows that more than five-sixths become lunatic through intemperance: a word which, properly translated, means habitual and irrational indulgence of animal passion.

The accurate reader will at once remark that to raise our proportion from a half to five-sixths, we have deducted the idiotic from the mass of lunacy in the community at large, but not from the lunacy of the Friends; to a very limited extent we admit the inaccuracy, but we are not guilty of it unadvisedly. The professional experience of Dr. Thurnam, on which we rely for the statistics of Quaker lunacy, supplies us with this remarkable fact:—

"Idiotcy and positive imbecility from birth appear to be of very unfrequent occurrence in the Society of Friends as compared with the general population of this country." And he gives a very satisfactory reason for it. "It is, perhaps, not improbable that many in this society who, by careful nursing, survive the period of infancy, and are merely distinguished by these slightest shades of mental weakness, would, under less favourable circumstances in the lower walks of life, in the world at large, have grown up as positive idiots; or that, with that delicate organization which distinguishes them, and which they perhaps inherited, would never have been reared at all."

We think that this is a sufficient authority for deducting the imbecile from one side of the proportion, and omitting to deduct it on the other.

So far, then, as we are supplied with data, it is clear that the experience of the profession confirms our theory. Insanity in the large majority of cases is induced by the excessive indulgence of those passions which God has given us as necessary stimulants for the support and propagation of our animal nature; and we have endeavoured to exhibit the actual working of the process by which insanity is thus induced, in the successive defeats of reason by the superior strength of passion when indulged. There will always be found, especially in the world of science, determined cavillers at a new principle, or more accurately, in the present case, we should say, at a new application of an old principle; we may, therefore, anticipate the question—What is here meant by the term "reason," or in what sense is it used?

This is a convenient opportunity for introducing a remark of value to our theory. The doctrine of perpetual internal conflict between right and wrong is of very ancient date. Cicero arrived at it by induction from philosophy alone. St. Paul, writing under holy inspiration, describes the conflict in very similar, but yet more specific language:—

"For that which I do, I allow not: for what I would, that do I not; but what I hate, that do I. If, then, I do that which I would not, I consent unto the law that it is good. Now then, it is no more I that do it, but sin that dwelleth in me. For I know that in me, that is, in my flesh, dwelleth no good thing: for to will is present with me; but how to perform that which is good, I find not. For the good that I would, I do not: but the evil which I would not, that I do. Now, if I do that I would not, it is no more I that do it, but sin that dwelleth in me. I find, then, a law, that when I would do good, evil is present with me."

The authors of our liturgy, partly on the authority we may presume of this passage in the Epistle to the Romans, have introduced a prayer in the collect for the first Sunday in Lent.

"Give us grace to use such abstinence, that, our flesh being subdued to the Spirit, we may ever obey thy Godly motions to righteousness," &c.

We seem warranted, not only by these passages, but by the whole tenour of revelation, in the belief that there is, not in any metaphorical sense, but in actual fact, a warfare carried on between good and malignant spirits, of which, for some mysterious purpose which it is not permitted to us to penetrate, humanity is the field; it may be that these hostilities extend to the whole material world, visible or invisible, or even to regions where matter is unknown; the strong presumption, however, is the other way, and that the spirit which we call sin, is allowed no wider range than the earth which we inhabit; for though, most assuredly, the great sacrifice offered for the atonement of man, would suffice for the sins of all created existence throughout the boundless realms of space, yet as our insignificant particle of matter called the earth was selected for the altar appropriate to the sacrifice, the inference is either that we are the most favoured or the most wicked of God's creation; or yet, more probably, that we are the only beings of limited existence who have dared to range ourselves with the fallen immortal spirits in their resistance to the Creator's will. Milton was a sound divine, no less than a sublime poet, and such certainly appears to have been his creed. But it is foreign to our subject to enter upon such mysteries, nor is the infirmity of human understanding equal to the inquiry. We advert to them only to remark that our analysis of the struggle between reason and passion, as a metaphysical thesis, is in perfect harmony with what revelation has deigned to tell us of the unseen warfare that obtains between immortal spirits. The conflict itself is, doubtless, the same, whether we choose to describe it by one name or another. St. Paul speaks of "evil" and "himself" as the contending parties, whom we designate as passion and reason. Our object has not been to start any new hypothesis, but to avail ourselves of the Scriptural truth as a clue to the explanation of that phenomenon which has so long perplexed us; where shall we fix the limit of man's responsibility as a rational being, in reference to the laws of society? It is with this view that we have attempted, it may be feebly and unsatisfactorily, to propound a somewhat novel theory, as to the actual development of this conflict by its visible effects on the moral and physical organization of man.

We proceed with our answer to the anticipated question, in what sense we use the term "reason."

Every animal, whether human or bestial, is endowed with certain properties for self-preservation and self-generation; in the lower orders of creation these properties are called instinct; man, as an animal, enjoys an instinct too, but in him the instinctive faculties are associated with powers of a far higher quality, with a view to preparation for a nobler and an eternal state of existence.

His instinct, apart from these higher powers, resembles the instinct of any other animal in its essential properties. It is perfect from his birth; it is not progressive, because it is incapable of improvement. Instinct guides him to the breast; instinct dictates the squalling of the infant as well as the bleating of the lamb; instinct makes him shun pain, and cry for protection from approaching danger; the infant will shrink from a dog or a cat as soon as his eyes are capable of observation; he fears entering a field where cattle are feeding; he shrinks from the touch of strangers, and will even hide himself on their entrance; he runs to his mother at the howling of the storm or the pattering of the hail; all this fear is instinctive, and shared by the infant with the animal creation. As he advances to puberty, other instinctive feelings are awakened, and are indicated by the same change of manner, appearance, and disposition, that mark the puberty of brutes.

But though his instinct is not progressive, those higher powers with which it is associated are; he is at an early age conscious of a will to obey or to resist his animal propensities: he cannot define this elective power, but he feels it: he is sensible of a freedom of action wholly independent of his animal nature: he marks the distinction between himself and dumb animals, not merely in outward form or in their respective objects of desire, but in volition. It may be restrained by circumstances, it may be fettered by parental authority; but still he feels and enjoys *the will.* This development of will in independence of necessity, is the ray which opens the bud of reason; what may be its germ is known only to the Creator who planted it. As it expands, it exhibits faculties of calculation, of deduction, and of anticipation. He finds these faculties subservient to his animal wants, which instinct explains, though it can no longer provide the means of gratifying, and here the range of uninstructed reason closes. If we could conceive a man abandoned at this crisis to absolute solitude in the steppes of Tartary or the wilds of America, and destitute of all means of information from social intercourse, it is probable that he would degenerate into mere animal existence, though gifted with more cunning and less instinctive sagacity than the ourang-outang. He would indulge to satiety when food was abundant, but if sickness followed the indulgence, his rationality might prevail to check the repetition, till long privation provoked to a second surfeit; if the pain of sickness had been great, instinct would step in to restrain him, and reason would suggest reserving out of the abundance for a future meal; but whatever aid he might thus derive from reason in self-preservation, self would still remain the sole object of his thoughts, and animal indulgence the single end of all his efforts. In such a case reason would not become enfeebled, simply because she would be subjected to no struggle: subject to no law but that imposed by instinct for the preservation of the animal, she could violate no obligation; and the stronger she found the animal passions, the more imperative would be her duty to contrive for their gratification. She would probably become the slave of passion when conscious of no responsibility to any other master.

But the development of will not only gives its first bloom to the reasoning power, but informs the juvenile logician that there is imposed on his action an artificial constraint under the name of law: his will prompts him to the gratification of his instinctive wants; his reason suggests the means of gratification; and, simultaneously with this newly acquired servant, he discovers that physical restraint impedes the freedom of his action, though not of his volition. First there is the law of the nursery; he resists and is sent to bed. Then there is the law of the school; he still resists and is whipped. The law of the academy follows; he still resists and still is punished, restraint being throughout associated with disgrace. At length, emancipated from all physical control, he enters on the world at large, and there finds that a double code of law is enforced; the penalty of a breach of the one being corporal punishment combined with infamy; the breach of the other being visited by disgrace and exclusion from his caste. Where education has been based on religion, he finds a yet more formidable check, and yet more dreadful penalties, though more remote.

Reason is thus exercised in early life by continued struggle, and gains strength by the conflict because she is assisted by physical and foreign discipline; while this continues she can do battle with volition and bring it into habitual subjection. The triumph tells to her advantage even on the score of gratification, for if less intense it is more certain in its occurrence and innocuous in its results. At length, however, this foreign aid is withdrawn, so far as regards immediate check; the penal consequences of indulgence to excess are removed to a distance, and reason and passion are left in an open field to "fight it out" as best they may. The will desires to remove all impediments to gratification; the faculties of calculation, deduction, and foresight soon devise a way, but they at the same time distinguish danger in the distance, which instinct cannot see, and to which volition will not give credence. If these faculties, which we conventionally express by the term "reason," retain the power given to them by habit, volition remains in subjection still; if the force of habit is relaxed, volition regains her early ascendancy, and the animal predominates over the intellectual; this ascendancy is at first transient. It is a part of our animal nature, and mercifully ordained by the Creator, that pain, whether of mind or body, is only a present sensation; man cannot long exist under the pressure of unceasing pain; as physical causes will always produce their physical effect, pain will follow the first transgression of temperate limits; while the pain continues, reason condemns volition for its folly, and resolves to withhold further aid to its gratification. The pain subsides, and soon ceases to be recollected in all its acuteness, or even to be forgotten altogether. A first offence entails no permanent disgrace, and reason begins to urge that she has overrated the distress of punishment: she has undergone it once, and it is not so severe in recollection as it used to seem in anticipation; thus she is prepared to yield more readily on her next encounter with volition.

It is another general law of pathology that the second attacks of the same disease are, in their immediate and painful symptoms, less intense than the first, where the complaint is not, in its nature, chronic, or proceeding from constitutional affection: cases are constantly to be met with where the reiterated recurrence of a local disorder, gives it an incurable hold upon the system, and yet the patient scarcely suffers pain amounting to inconvenience, though in its earliest stages the pain was acute.

Something of this kind obtains in excessive self-indulgence: smoking gives a familiar illustration; the first time that the fumes of tobacco are inhaled to even a moderate extent, most distressing sickness follows; the second time, if the interval is long, the same result will follow, but the nausea will be less and of shorter duration: after three or four experiments, this painful derangement of the stomach is no longer felt, unless the indulgence has been extreme; and eventually a man will smoke all day unconscious of any inconvenience. Many other morbid affections would admit of similar illustration, but for obvious reasons we forbear.

Reason, when defeated in her second conflict with volition, again suffers the penalty of pain, but in a less aggravated form: the effect of intemperance is the same in character but less in degree, and its recollection less admonitory, after every successive trial; the seeds of chronic disease and permanent debility are abundantly sown, but the painful paroxysms that at first were instant in their sequence, are no longer felt; thus the penalty, though still inevitable, is more remote, and reason, not sustained by immediate apprehension, is more and more enfeebled in her resistance.

We have thus far only described the struggle as it might obtain equally in the hypothetical case which we have put, of an utter outcast from society; a struggle between volition and reason, where pain is the only restrictive penalty; and we have adopted this simple form because it affords a plain view of its nature and progress. Though the laws of society interpose other restrictive penalties, and so far have strengthened reason for the conflict, the tactics of the warfare remain the same whether we place man in a social or solitary condition.

The inference which we are entitled to draw from these premises is, that it is exactly in proportion as self-control is rendered habitual by early training, that reason is enabled to retain her powers in health and strength through life. The force of habit must be added to the force of reason to keep the volition of the animal in constant, unvarying subjection. It must not be supposed that we overlook or depreciate those better motives that religion inculcates, or that all-powerful support which the sincere Christian derives from the grace of the Holy Spirit; we are considering the subject in the only light that befits a scientific journal; as connected with metaphysical inquiry into the structure of man as an intellectual animal, gifted with instinctive passion on the one hand, and with self-controlling faculties on the other: a free agent as regards his volition, yet restricted by physical and social responsibility as regards his acts.

Another objection which may be raised to our theory appears very plausible at its first enunciation. How does it occur that when the proportion of lunatics among the higher and middle classes is so large as 5000 to 18,800, or more than a fourth of the whole number, the proportion of criminals among the same classes is so extremely small as scarcely to amount to an appreciable quantity? It might be inferred that where so many are found incapable of subjecting their passions to reason, crime would abound among them to a much greater extent, if legal criminality and inordinate self-indulgence are identical in their origin, their progress, their objects, and their results; crime is, with rare exceptions, confined to the pauper class; lunacy, having regard to their relative numbers in the population of the country, is nearly twice as prevalent among their superiors; assuming, that of the entire population in 1841, half a million will represent the higher and middle classes, it follows, from the report of 1847, that their liability to insanity is in the proportion of one in 500, while the corresponding liability of the rest of the community is only one in 840: but the proportion of legal criminals is at least 100 to 1 against the pauper class. We have no data to estimate this latter proportion; it may more likely be 500 to 1, but it is enough ground for the objection to take the lowest estimate. We promised to advert to this topic in our note at page 185.

Instead of feeling it to be incongruous with our theory, we think the fact goes far to sustain it; we have already alluded to it in a former page, but only cursorily. It is almost a proverbial remark, that our laws are made for the poor and not for the rich; and there is necessarily truth in the remark, though not in the sense of vulgar declamation on the hutsings. Food and warmth are the most pressing of our natural wants, as well as the most frequent in their occurrence; the cravings of appetite, whether in ourselves or in those who are dependent on us, must be satisfied at all hazards, and there is only an inferior degree of urgency in the necessity for clothing: hence the pauper is so often tempted to appropriate the property of others, not for excessive gratification, but for the indispensable nourishment of his animal nature and in strict obedience to animal instincts, that not only is legislation continually at work for the protection of property, but our judges, for the most part, reserve the severest penalties of the law for theft or fraud, visiting crimes against the person with comparative lenity. Every assize and every quarter sessions produces instances of transportation for felonies of this class, in absurd contrast with imprisonment for a few months for manslaughter, or assaults with intent, &c.. The life of a man or the honour of a woman is often ludicrously weighed against a loaf or a yard of broad cloth in our scales of criminal justice, and kick the beam: a child of seven years of age is, at the moment we are writing this, imprisoned in Knutsford Gaol on a charge of stealing a mug of the value of a penny! This extreme severity of the law and of the judicial caprice with which it is administered, not to mention the enormous expenses of prosecution, induce many to overlook the injury they sustain; and thus the pauper who begins by stealing to supply actual want, is emboldened by impunity to steal for gratification of his animal passions, ultra the necessity of his case: reason in vain points to consequences when experience proves impunity, and thus she loses all the aid of restrictive penalties in resisting the assaults of passion. We shall presently give a narrative pregnant with illustration of this, and shall have further occasion to advert to the same topic in considering remedial measures.

The higher and middle ranks of life are not exposed to similar temptation till a long career of extravagant indulgence has reduced them to actual want; even when their own resources fail them, the benevolence of friends, or more prosperous relatives, steps in to save them from actual destitution. Thus the insanity induced by intemperance will often overtake them before they violate any law but that which is imposed by the social code of decency. Even when reason is rapidly declining, she will retain sufficient restraining power to prevent a man incurring unnecessary hazard, when he can as easily obtain satiety of gratification without exposing himself to legal retribution. The loss of caste is also a restraining penalty, strongly operating in aid of reason among the higher orders, but it is unknown to the low-born pauper. Their sensual excesses, therefore, generally take a direction which entails no public ignominy. As for disgrace in their domestic circles, it is covered by affection, or, at the worst, retrievable by amendment; but whatever be the direction of a pauper's passions, if gratified at all, they must be gratified at the expense of others; hence, the penal law, though made alike for all, seems, by its almost exclusive application to himself, to be intended for him alone. Were the pauper criminal a man of wealth, he would become insane before he is marked a felon; but being a pauper, he becomes a felon before he is ripe for the asylum; abridgment of opportunity, and the discipline of a prison, preserve him in the incipient stage.

In confirmation of this view of the subject, it may be remarked, that in the comparatively few cases in which men in the higher walks of life become amenable to the law, it is usually found that it is for some of the offences that fall within the description of malicious violence to the person. When the passions of a malignant type are those habitually indulged, such as anger, jealousy, revenge, or the coarser sensualities, reason, though aided by all the restrictive penalties of the social code, becomes subdued in his case as easily as in the lowest class; murder, and manslaughter in all its variety of guilt, violence to women, and even vindictive injuries to property, are crimes not confined to the pauper class; though far more frequent among them, simply, because reason has not been fortified by habits of self-control, and strengthened by education.

Nor is it less important to remark that, though in common with the rest of the world liable to other predisposing causes, the Society of Friends is exempt from intemperance as an inducement to insanity, and equally exempt from appearance in our criminal courts. Some spurious offsets of their body have now and then been arraigned, as, for example, Tawell; but it has always been found, on inquiry, that in these cases the dress had only been temporarily assumed, or the membership of very recent date. That mental powers and peculiarities are often transmitted from generation to generation, is undoubtedly true; but it would be extravagant to infer from this, any peculiar idiosyncrasy distinguishing the whole sect. In truth, those who have been admitted to terms of intimacy with them, a privilege which we have often enjoyed, know very well that, as a body, they yield to none in strength of passion, or generous warmth of feeling. Prudence, certainly, wears with them a severe aspect, but it is a self-controlling, not a freezing prudence. Where passion is right in its direction, and noble in its object, no man will give the reins to it with more freedom than a calculating and prudential quaker.

And, to adopt the habitual phrase of one nearly allied to them, the late Sir Fowell Buxton, who was not less sound in his argument than resolute on his point, who never maintained a point where argument was wanting, nor wanted argument when the point was generous and good, "the sum of our argument is this,"—man, as an animal, is endowed with instinctive properties for self-preservation; but, created for responsibility, volition is given to him that he may be a free agent, and certain faculties that we designate as "reason," are also given to guide his acts by reference to their consequences. His animal instincts impel him in a right direction, and his volition, partaking of animal instinct, carries him to excess; reason's function is to restrain volition in its tendency to excess, by the fear of penal consequences injurious to the animal nature. An unceasing conflict is thus maintained between two antagonistic powers. There can be no compromise between them; one or the other must succumb. If reason habitually triumph, she retains her seat till death; if she habitually yield, at last, she abdicates from debility and exhaustion. We have shown the identity of crime with insanity, in its progress, its objects, and its results. We have proved that intemperance, in its largest sense, is the predisposing cause of both, except in such lunacy as in its development betrays the acknowledged signs of local disease or organic malformation; and we have drawn this proof from statistics published by authority, or sanctioned by large medical experience. We now propose to proceed to the consideration of the remedy. But to effect a break in our elaborate dissertation, as our judges retire for half-an-hour at one o'clock for a glass of sherry, after a speech from Thesiger or Kelly, we will here introduce our promised narrative, in the same homely dress of grammar and orthography in which we have received it. It is extracted from an official report of the Rev. I. Clay, the chaplain to the Preston House of Correction. We have erased some portions of it not applicable to our subject. It will be found to contain a most striking picture of the graduality of intemperance in generating crime. It is the autobiography of a coiner.

"I was born in 1800. My father was an honest and an upright man, but he was much afraid some misfortune would occur to me, and his words has proved true, for I have gone through more than all my sisters and brothers put together; but I have earned the most money. With all my earnings I am now by far the worst off; all my sisters and brothers are in very creditable circumstances, while I am now within a prison walls. My father left seven children. We were all sent to live with my grandmother, but we were all soon separated. I was put to live with a man at the place where I was born. He was a man that I believe never attended any place of worship, except upon the occasion of a wedding or burying; but I often heard him and his mates boasting which had the best game cock, and which was the best fighter. He had eight brothers, who were all fighting men: they were all hand-loom weavers, and they kept a snug farm. It was about the time that peace was made, after the battle of Waterloo.

"At the beginning of the week, for two or three days, it was drinking, fighting, and cock-fighting, card-playing, &c. His wife died, and we were then removed to his parents. We were about twenty, all in one family. There I learned to know what it was to be without parents, for I was under the control of the whole family: if I disobeyed any of them, I was rewarded with a kick or a blow. One Sunday I went to see my grandmother, and I had four or five cuts on my forehead and ears, some of them bleeding at the time; so my grandmother got me into the factory, Lower Darwen, where I was bound apprentice for seven years. I never was so happy as I was at that time, though I never saw anything like religion exercised. The master was not content with the bell-ringing, but used to come to every door in the morning to call his workpeople up; and I have known us to work until sometimes eleven o'clock at night, and on Saturday nights occasionally until twelve; and after that time he would take all the men to the public-house and give them plenty of drink, and they would continue drinking until the morning. On the sabbath they would lie in bed all day.

"I served my time honestly, and I had not a bad master after all; but he was a heavy drinker. In his mill a schoolmaster attended twice every day, to teach all the hands that had a mind, and from him I got most of the little learning I am possessed of.

"I was married, August, 1824. We had 33*l.* and a few shillings, and all things went on very smoothly for a long time. I still kept in work at the same mill, and we got on very well until the mob attacked it in 1826, and broke all the power-looms; so I was six months without work; so I went over to Wigan, and I had 10*s.* a week for looking over the other spinners, and I was getting upwards of 2*l.* per week off my own wheels, and all this time I never got to drinking; but soon after I got to like drink, and made a practice of going every Saturday night with my wife and the other spinners, till at last I got to taking whole days. When I first started to drink I had above 200*l.* in money, and as good furniture as any working man need have. We had been married above nine years before I began resorting to these places, which have been my destruction. I *was* a happy man. I used to have my children well clad, well fed, clean, and comfortable, and my wife the same; and I could go to a place of worship on a Sunday. When the labour at the factory was over, I used to work two or three hours at home for my own pleasure and advantage. I had a lathe, and got many a crown for making chairs, &c. I carried on drinking for a long time, still going longer and worse, until my money began to lessen very fast; so I began to be more steady, and did not drink much for near twelve months. I earned that time, with what I got in the factory, upwards of 3*l.* every week; so in one year I saved between 70*l.* and 80*l.*, besides maintaining a wife and four children. When I think on those days, and my being now confined in a prison, and that same wife likewise, and one of those dear children that we used to take such delight in, confined within a few yards of me! And what can be the cause of this do you think, seeing that my former circumstances was so prosperous? I can explain the cause in a very few words:—neglect of the sabbath, drunkenness, and bad company; but drunkenness, I do affirm most solemnly, has been the cause of all the other evils. But to my story. I worked at that mill twelve years, until our master's health began to decline, and the mill began to make short time; and what little the mill did run I was not to be found, for my time was the most employed in the public-house; and this was the time I began to ruin myself; and, still worse, my wife commenced drinking, and then all soon went to ruin. At our master's death the mill stopped altogether, after which I left Wigan. I had been at Bolton and taken a beer-house, and had promise of work at Mr. Bolling's. I took the beer-house, thinking as my father and mother-in-law had nothing to do, they might make a little by selling beer. We might have done very well had I been steady, for I got a very good pair of wheels, and the house I had taken was convenient to the factory, and we got a good deal of custom: they came at night when the factory was over, and we would let them stop until twelve or one o'clock in the morning, cursing and swearing, and me, and perhaps my wife and her father; and no one but the old woman to fill, and perhaps twenty men drinking in the house. I have slept in my clothes all night, and have had to go to the hot factory at half-past five in the morning, and the spinners, perhaps four or five, lying on the floor, they were so drunk. As soon as I could get them up to go to work, they wanted more drink; and we would sometimes take five or six quarts to the factory; and as soon as we could we would get all our big piecers to spinning, and we would creep out of sight of the over-looker to drink, so that at breakfast time we might have another fetching; and this was the way we used to go on, so I got the name of a regular drunkard; and the manager told me, if I did not give up the beer-shop he should be obliged to acquaint the master, for, he said, all the spinners were getting drunkards. At this time my wages, on an average, after paying for rent, milk, and beef, was about 32*s.* per week clear; but I found that the hot factory and so much drink was causing my health to decline; so I left Bolton, and went to spin for Mr. Side-bottom, in Derbyshire, and the old people came to live with me, and we were very comfortable. I was getting much less money than at Bolton; but we began to mend; for I began to joiner a little at night; and it was well I did so, as the mill went on short time for above six months; and as there was no one who kept a joiner's-shop, I got as much work as I could do. Just at that time my wife fell sick, and continued so above twelve months; and then one of the children died. I took much to drinking through the death of that boy: but drinking was a sinful folly; and if I had the same to do now, I think that instead of flying to the alehouse I should fly to the house of God. At this time I was getting but small wages comparatively—about 1*l.* 2*s.* or 3*s.* per week, and my three children used to get about 10*s.* per week between them. I was there about nine months weaving, before I got a situation as overlooker. I was at that place but a very short time before my mother-in-law died; and that was the worst shock I had ever experienced; for we had six children, and my wife was not able to attend to them, on account of losing the use of one hand during her sickness. I think after the death of my mother-in-law, I was more negligent in my duty to my family than I was before; and I began to drink and neglect my work; sometimes off my work a week or a fortnight drinking; and the last time I was off, the master told the manager I must not start any more. With that I took about 2*l.*, and went to Ashton to see for work; but instead of looking, I went straight to the place of drunkenness, where I knew I should find plenty of company that were spending their money and neglecting their families, like myself. I could, at that time, have gotten near 2*l.* per week with comfort, if I had been a steady, sober man. I went to Ashton on the Wednesday, did not return home until Sunday morning, with not one penny in my pocket, and 1*l.* in debt.

"I went to Blackburn to see if I could get work, but when I got amongst my old friends, I could find but little time to look after employment for drinking. I was three weeks at Darwen and Blackburn, and had come near forty miles for work, but did not ask any person for work, though I had it offered me if I would bring my family. At last I did, and we were getting on very well, but my wife took to drinking very heavy; she had got acquainted with a class of women that made a constant practice of drinking: often when we came from the mill have we found her drunk in bed, and nothing prepared for us to eat, and having at that time four children unfit for work, who were destroying and wasting the provisions we had to live on, for the want of a mother to look after them. Bad as I was, I never lifted my hand to strike her in all my life, for I was aware that if I had been a sober man, my wife would never have been a drunkard; so I began to think it would be the best plan to leave the town altogether, to separate my wife from her drunken companions; so we went to Bolton, and I made the acquaintance of a regular set of drunkards, who would do almost anything to obtain money to spend in drink. I was always ready for a spree, and they were never short of money, though they were scarcely ever seen to work. One of them was a very good shoemaker; his name was N. S.; the other was a labourer, and went by two or three different names. One day, as they were all drinking at my house upon a Sunday, I said to them, 'I do not know how you men scheme it, for you are never without money, and you work very little.' 'Ah,' said one of my new pals, 'there is none who will work except fools and horses;' and I said, 'I should be very glad if they would teach me, for I was getting very tired of working:' and they did learn me, to my sorrow. A very short time after they told me the grand secret, that they got their living by making and paying bad money; and they told me they could get as much money on a Saturday night as I could get in a whole week by working. So it was agreed they should get some ready by next Saturday, so we all three set off to Tyldesley Banks. We went through Straight Gate, and came back through Chowbent, and we paid that afternoon and night about 4*l.* They declared I was one of the best payers they ever saw; and no doubt I did my part well, for they gave me drink, and drink possesses me of a false spirit. They gave me 10*s.* for my share in that afternoon's work, and 10*s.* in bad money; and I paid that ten on Sunday in good time; but when they got to know I paid it in Bolton, they said I must not pay any more, for it was very bad to pay in the town we reside in, for it may cause suspicion, and that sometimes caused inspection. I kept company with those men upwards of twelve months, making and paying more or less every week. I had left off work long before the year end, and followed nothing except the bad money trade and drinking. But I had many narrow escapes from the police. They were both taken before me. While we were all drinking in a liquor-vault in Bolton, in came three policemen, and took both of them, but they took no notice of me; so I went home as soon as I could, and removed all that was in my house out of the way, for I had about 4*l.* or 5*l.* of bad money there at the time, and three moulds. They were both committed to Kirkdale for the assizes. At their trial I provided them both with counsel, and they both got acquitted. When they came back they soon commenced their old trade again, and wished me to join them; but for three or four months I had nothing to do with them as regarded the bad money, although I went with them drinking, until at last I joined them at the same game again, and I was not long with them the second time before I was taken prisoner. I got with my old mates again, and I asked them what I must give them to make me a few pounds, and N. S. said they would make me 10*l.* for 10*s.*, if I would find the metal, and they would come to my house to make it on Sunday morning: Sunday was the best day for making, as the police were always engaged in other business; and I was to buy a dozen of Dixon's best Britannia metal spoons. We all got quite drunk that night before we parted, and it was the last time we three got drunk together, for in a few weeks we were all three in prison, myself first. I was taken in Bolton, in a drunken state, with about thirty base sixpences, shillings, and half-crowns in my possession, for which I got twelve months. When I had served my twelve months, I found my wife and the younger children in the workhouse; those that were old enough to get their own living had left her, so when I come out I had no home or friend to go to, for all my relations had turned against me; so I went to Bolton, and was well received by N. and a few more of the same sort; there was plenty of drink, and plenty of bad money. On Monday morning my wife got a pound from the overseer to leave the workhouse, with which we bought furniture, and I got work for myself and as many of my family as was able to work. We had not been above ten days in work, before the police came to the factory and told the master we were a set of bad money makers; so the master sent for me and told me we must get another situation, as we had been very badly reported to him, so we were all without work again. So I asked myself what must be done now, as it will be of no use my trying to get work in this country; so I said to my wife, 'Go and get the children in work if you can, and I will try the bad money system again, in order to get a little to leave the country altogether, if I can.' Trade was very bad at that time, and we could get no work, so we continued paying until my wife was taken with Mrs. Preston. They both pleaded guilty, and Mrs. Preston got six months in Preston house of correction, and my wife three months. So I was left with eight children for that time, five of whom were not able to work, had there been any for them; but there was no work at that time for one-third of the people in Lancashire, as almost all the factories were either standing or running short time. So I went to the relieving-officer and before the board of guardians; but the workhouse was full, so they gave me a paper to get soup and bread, and I was very thankful for it; but all we got from the charity was not sufficient for my family, for we were nine in all: how were we to live? To carry on with bad money was very dangerous, and owing to my wife being taken, the police came several times to my house to search, so I got a few shillings from the relieving officer to begin barbering; but I got very little custom, so I was determined to see my children starving no longer, for we were whole days and never tasted food, so I went to Bolton to see if my old mate N. S. could do anything for me. When I got to Bolton, N. had got twelve months in the New Bailey Prison, Manchester, for buying stolen goods, but his wife was very glad to see me, and gave me 5*s.*, and put me about 5*s.* or 6*s.* worth of food up to take to my family, and she said she could let me have some metal that N. left. It had been stolen, she thought, for N. had told her she must keep it in the house. So as I was waiting of this woman raising the plant, I went to see my acquaintance old J. O. and K., as they had a quantity of base coin in plant, and they said if I dare pay any I must have a few half-crowns or shillings; so I took a few of each sort. O. said if I had a mind he would come to Blackburn with me and stay with me until my wife came home, for no one knew him in that quarter. So I was glad of his proposals, and we returned together to Blackburn, having got the metal from N.'s wife, and about six pounds in base coin. When I got home my eldest daughter was waiting up for me, so I went and fetched a gallon of the best beer and a pint of rum. Honesty is the best policy. Yes; for whosoever defraudeth his neighbour shall be found out, as my present situation in prison plainly shows; my wife is in prison, and my oldest son also. I am the father of nine children, or was so when I came here, but up to the time I write this I have heard nothing of them; we have had thirteen children; I am upwards of fifty years of age, and my constitution much injured through imprisonment. We have completely lost our character. Had I done what was right in the sight of God and man, my children might have proved a blessing to me and my wife in our old age; and I am convinced had I done as I should have done, that one or two of those children now in their graves would now have been living, for my wife, through having to look after me, and being in trouble, neglected the little ones. It is my sincere wish, as a penitent, that those who read this narrative may profit by it, and I wish the reader to compare the commencement of my married life, when I never frequented the public-house, and was happy in my own house, with the amusement of joinering or birdcage making, with that which followed. The publicans got so little of my money that we had always credit sufficient, and no person had to say, if they came to see us, we were short of anything. There could not be a more happy couple than we were; we never had a cross word for years after our marriage, and as to blows, I never struck my wife in all my life. Ah, but since I had to do with that destructive and ruinous drink and base coin, I have been the most unhappy man living."

As an example of the tendency of one form of intemperance, this tale is conclusive. Similar gradations will be found to exist in all its varieties, whichever may be the passion habitually indulged. The crowning act of irrationality or crime is no index to guide us to the diseased passion; for though, in our unsophisticated state, each has its peculiar province in stimulating to acts necessary for gratifying our instinctive wants of self-preservation and self-generation, yet passion begets passion in such large variety, that at last we cannot distinguish the progeny from the parent stock, or appropriate to each its peculiar functions in the animal economy. We cannot trace back the crime or the folly to its motive, from its own peculiarity of feature. Tawell committed murder to conceal the shame of a minor offence. Rush committed murder from revenge. Burke perpetrated the same crime as a trading speculation. Eugene Aram (divesting the story of Bulwer's romantic colouring) from a combination of vanity and cupidity. Thurtell and his associates, in aid of gambling propensities. Not one of these cases was marked by drunken habits. Not one of them was free from the visible taint of irrationality. Yet, apart from the circumstances disclosed on trial, who could have assigned an adequate cause for their respective crimes, except in the habitual and intemperate indulgence of some favourite though latent passion?

It is in this comprehensive sense that intemperance is included among the predisposing causes of lunacy for which a remedy should be provided.

This remedy is to be found in a revision of our system of national education, so as to give it more extensive operation, and a revision of our criminal code with a view to its greater efficiency: the first of these remedial measures is sufficiently obvious; the last will not be less acknowledged when we examine closely into its present administration.

It would be waste of time to argue out the truism that self-control is the invariable fruit of early education.* Of course it is; it is at once

* There is one singular exception to the general rule most ably established by Mr. Fletcher, in his "Moral and Educational Statistics," that crime decreases as education advances. This exception is to be found in the county of Cheshire, "which," observes the learned author, "stands alone in its inky blackness in every moral characteristic, except in regard to instruction, in which, unhappily, it bears a more favourable tint than nearly all that surround it."—*Stat. Soc. Journal*, vol. xii. p. 191. From our personal knowledge of that part of the county which contributes its undue share of crime, we can explain this seeming contradiction to Mr. Fletcher's doctrine. He estimates the amount of crime by the number of commitments; a very fair criterion, because every commitment is strong presumptive evidence that a crime has been committed, though the guilt of the party accused may be doubtful. In the case of Cheshire, however, it is not so; for in that large and densely peopled district, of which Birkenhead is the centre, and which, from its vicinity to Liverpool and the coast, is probably the most prolific of crime, the bad system prevails of paying no salary to the magistrates' clerks, and of remunerating them for their professional services by the costs of the prosecutions. As county justices are rarely able to move a finger except under the guidance of their clerk, there will never be any lack of commitments when the clerk has no other treasury to look to for remuneration!

the end, and the means of accomplishing its own end. Yet the deficiencies of our national system are such that one might almost suppose that this admitted axiom were still to be explained to the conviction of our legislators! They certainly have adopted as a general principle of legislation a maxim so antagonistic in its character, that the progress of education among the lower classes is almost arrested by it. Compulsory adoption of the opportunities of education is considered incompatible with the just liberty of the people. Hence it is left optional even to those who cannot appreciate its advantages. If it could be proved by a comparison of the statistics of crime and education, that the latter is practically inefficient to check the former, some weight might be allowed to this objection, founded on principles of civil liberty. This comparison has been attempted on very insufficient data, as regards the statistics of education, it must be confessed; still the result corresponds with our views. In some papers read to the Statistical Society by Mr. Fletcher, and which appear in their quarterly journal for August, 1849, the learned author states as one of the "conclusions" at which he arrives :—

"The conclusion is irresistible that education is not only essential to the securing of modern society, but that such education should be solid, useful, and, above all, Christian, in supersedence of much that is given by the weakest of the day-schools, and attempted by the most secular of the Sunday-schools."*

It is a "conclusion" of common sense as much as of statistical calculation. But the objection is destitute of the foundation on which it is assumed to rest: compulsory education is no violation of our free constitutional principles; so far from it, that instances are not wanting in modern times of the parental authority being superseded, even in the highest ranks of society, by the judgment of our courts, when that authority was wanting in providing suitable education for the child. The Wellesley case is familiar to every lawyer, and almost to every layman. He must be a very subtle logician who can suggest any difference that property makes in the principle, though it supplies the only means of bringing such questions within the jurisdiction of the Court of Chancery. If it is competent to that court to take its wards out of the paternal control with a view to their proper education, it cannot be foreign to the duty of Parliament to exercise a similar discretion as

* We cannot quote from Mr. Fletcher's papers without acknowledging the obligation he has laid upon the public by his talented and elaborate researches. These papers contain a mass of most valuable information, ingeniously classified and arranged: we do not wish to qualify our commendation, if we add the expression of our regret that they are not prepared with more attention to perspicuity and style—an error common to too many dissertations of the learned upon statistical subjects.

regards the infants of the realm, who, in common with their parents, are protected by its wardship.

It is not essential to our theory to go into the controverted question of educational principle; whether exclusively secular, or partially religious, all education must be necessarily based, more or less, on self-control. It is upon this, and on this alone, as an habitual discipline of the mind that we insist, as indispensable to sustain reason in her daily conflict with the turbulence of passion: a system of education that adds to other restrictions on excess, that which in well regulated minds is the most powerful of all, the apprehension of the divine wrath, is of necessity preferable to any other; but it is the discipline of education which we invoke, as the most efficient of moral means to restrain every form of intemperance that enfeebles the power of reason. A lamp-lighter and a letter-carrier are equally exempt from gout, so far as exercise will save them.

Returns of the schools of all descriptions have, we believe, been for some time in preparation; if made, they are not yet accessible to the public. We must content ourselves with the information we can derive from the blue books, entitled "Minutes of the Committee of Council on Education," relating to those schools which receive assistance from the parliamentary grant. It is not probable that the more general returns in preparation will prove more instructive on the particular points to which we propose to draw attention.

We collect from these Minutes for 1850 and 1851, page 156, that provision is made in England for the accommodation of 622,828 children in our national schools, not confining the term to those which belong to the Church of England. Considering that by the census of 1851, the population of England and Wales is 17,922,768, this would seem to be a very inadequate provision; but when we add that it appears from the same authority, that only 241,836 children habitually attend these schools, and that estimating the number of those who do not attend, by the proportion of absentees in Wiltshire and Berkshire (the only two counties of which the number is given), there are at the least 357,000 who never approach them, it will be probably thought that the provision is ample for the existing emergency, and that we must look elsewhere to account for the ignorance of the lower classes.

The reader will refer to these two books in vain for our authority for this calculation, without explaining the manner in which we arrive at the result. He will find annexed to the report of each district-inspector a table giving the number of children in habitual attendance. These tables include thirty-eight of the English counties and the Isle of Man. By adding together the numbers indicating the average attendance in each district, including the Roman Catholics, and such of the Dissenting schools as participate in the grant, we obtain a total of 241,836; and by comparing this with the proportion of absentees in Berkshire and Wiltshire, estimated, at page 141, to be 46,611 to 31,943 of labourers' children, we obtain, omitting the fractional parts of a thousand, 357,000 for the non-attendants. This is, of course, a loose calculation, but it is the best that can be made from any statistics already published, and it is sufficiently accurate to warrant our inference that the ignorance of the million does not arise from the want of opportunity.

Whence then does it arise? The experience of an anonymous writer is not of much value; yet fifteen years of that experience, in very different localities, enables him to offer a very plausible answer to the inquiry.

Poverty compels selfishness. It is not only uncharitable but absurd to suppose that the pauper parent is wanting in parental affection; for that affection falls within the class of our animal instincts; but when employment is precarious and food uncertain, all other considerations must yield to the necessity of securing the daily meal. An infant under six is an incumbrance, for he contributes nothing towards the common maintenance, while he monopolizes the mother's time; he is, therefore, gladly sent to the infant school; it is worth a penny a week to get rid of him during the hours of labour; but when he attains the age of seven or eight he can help to clean the house and watch his younger brothers and sisters, if he can do nothing else, and the mother thus gains two or three hours a day for profitable labour. At the age of twelve or thirteen, even the child can earn a weekly shilling or two in aid of the common purse. Were his own interests alone to be considered, he would be, far more wisely, kept at school, and especially at that critical age when the strongest passions begin to show themselves; but this cannot be, for "he must earn something." Such is the answer we have received in innumerable instances to the question "Why not send your boy to school?"

When the lad attains adolescence, he is quick enough to perceive that his weekly pay exceeds his share of the domestic expenditure, and at eighteen or nineteen he throws off the yoke, and, if he does not improvidently marry, he spends his money in the beer-shop, and begins that system of irrational self-indulgence of which we have been tracing the progress to crime and insanity. We have at this moment under our charge a pauper school, nominally consisting of some 150 children; from causes such as we have described, it is impossible to secure an average attendance of more than half that number, even upon Sundays. Personal expostulation with the parents is not wholly thrown away, for we have extended the average from forty to eighty-three by this means; yet, beyond this, advance seems impracticable.

We are fully sensible that in all this we are stating nothing new. There is not an individual that has personally exerted himself in the formation of parochial schools who cannot quote the same experience; but we imagine that it will surprise many to learn from the statistics of intemperance, what are the physical results of such a well known system.

In the Journal of the Statistical Society, for September, 1851, there is a paper by Mr. Neison, giving a comparative view of the mortality in persons of temperate and intemperate habits, at all ages. From this paper we learn that, at the early age of sixteen to twenty, the mortality in England and Wales, of the intemperate to the temperate is as 1·8 to ·730! and from the ages of twenty to thirty, as 5·1 to ·974! Is it possible to attribute this vast disproportion to any other cause than the early emancipation from all control that is the unhappy lot of more than half the labouring population?

The same authority, and writing, be it observed, on official data, namely, the returns of the registrar-general, supplies us with another result directly bearing upon our thesis.

From the age of twenty-six to thirty, delirium tremens is the cause of death in the proportion of 7 to 1 of nearly every other disease; from thirty-one to thirty-five, in the proportion of 10 to 1; and from thirty-five to forty, of 16 to 1; and, generally, diseases of the head are the cause of death in the proportion of 97 to 82 of respiratory complaints, and to 83 of diseases of the liver.

We have failed in discovering any average of the age of criminals, calculated upon authentic data. It varies between twenty-one and twenty-four. In the absence of correct information we lay no stress on this circumstance; but it seems worthy of remark, that for the three years, 1845, 1846, and 1847, the average number of commitments tallied very closely with the number of lunacies. We have already mentioned that the latter amounted to 26,516, while the former amounted to 20,698. —*Stat. Soc. Jour.* vol. xii. p. 207. This is easily explained by the axiom that like causes produce the like effects; the excess on the side of lunacy may be accounted for by the fact already noticed more than once; that intemperance, where it leads to legal criminality, is often arrested in its progress by the imprisonment of the offender, while it proceeds unchecked by the abridgment of opportunity, so long as its irrational excesses keep within the pale of the law.

When we advert to the revision of our criminal code as the other remedial measure, we limit ourselves, of course, to such an amendment of it as may render its restrictive power on the intemperance of passion more stringent; we suggest, as moral jurists, not as law reformers; all punishment has, or ought to have, in view, self-restraint, as regards the offender, and example as regards others. The lax and capricious administration of our criminal law deprives it of much of its efficiency in both these particulars.

The distinguishing trait of irrationality is recklessness of consequences, while good sense, and the self-denial which it inculcates, are equally marked by a prudential regard to consequences; the more speedy and certain these consequences are, the less is the effort of self-denial required. Even the habitual drunkard will not drink a tumbler of brandy at a draught, for he has sense enough remaining to know that it is immediate death. Burke, or his disciple, Bishop, would not have murdered a man for the value of his body, had instant detection and Lynch law been inevitable. The criminal of every class speculates on impunity, and he speculates with more chances in his favour than is commonly supposed.

First, he has the chance of escaping detection; this is not inconsiderable where there is no organized police. From the narrative which we have extracted from Mr. Clay's report, some estimate may be formed of the extent to which a man may go in his career of crime, even where detection is apparently most easy.

Then, if detected, he has the chance of escaping prosecution; a man's public spirit must be very great indeed, who will incur the cost and trouble of prosecution in ordinary cases. To waste three or four days in awaiting the pleasure of an unpaid magistrate, to travel some forty or fifty miles to the assizes or quarter sessions, there to be detained for three or four days more, and to pay travelling expenses for himself and half-a-dozen witnesses, hotel expenses, and legal expenses, and be allowed about a fifth part from the county purse, is a sacrifice which a man may, in his simplicity, make once, but he never will again. The culprit knows all this as well as a county magistrate or his clerk.

Then, if prosecuted, he has the chance of being acquitted, either from some legal oversight, or the sentimentality of a jury. The value of this chance may be guessed from the fact, that out of 28,833 commitments in 1847, not less than 7251 were acquitted or discharged for want of prosecution.

And lastly, if convicted, he has judicial caprice in his favour; for out of the number of 21,542 that were convicted in the same year, not less than 15,499 were sentenced to less than six months' imprisonment. If they chanced to be the winter months, this punishment would be a boon to nine-tenths of them.

With such an accumulation of chances in favour of impunity, and with such a remote possibility of severity of punishment when it happens by any accident to follow, what becomes of the self-restrictive tendency of our criminal law, where the social condition of the culprit is so low as to render inoperative the fear of losing caste? Nor is the exemplary force of punishment less endangered by the capricious inequality of its infliction.

The million derive their impressions of legal obligations by experience of it in the persons of others, if not of themselves. They have no instruction in the principles of jurisprudence, or of ethics; no access to tuition, either oral or written, on subjects like these. Their estimate of the criminality of excessive self-indulgence is formed by its visible effects. They restrain an intemperate propensity because they see the drunkard revelling in misery, or the thief carried away in handcuffs to the cells of a prison. Such plain matter-of-fact lessons as these are sufficiently intelligible, and their impressiveness ought not to be diluted. But what must be the confusion created in their minds as to the heinousness of crime, or the guilt of excess, when in one town the poacher is visited with more severity than the burglar, and in another with less? When the bigamist is imprisoned by one judge for a month, and by another for a year? When the perjurer is fined to-day, and transported to-morrow? When the manslayer is sent to Norfolk Island from the Old Bailey, and to the treadmill from Aylesbury, or possibly discharged with a reprimand, at Chester? What can they know of "extenuating circumstances," who never hear the trial? or of the subtle distinctions of time and circumstance, that mitigate the penalty, though they affect not the legal classification of guilt? Our diversities of judicial administration are yet more singular. A cow may sometimes be stolen with more safety than a cabbage; a banker's parcel at less risk than a pocket handkerchief; or a rib broken at less expense to the assailant than an eye blackened! More strange still, a man may be punished in one place for an offence of which he has been just acquitted by the jury; and another, tried elsewhere on the same charge, be discharged scatheless, notwithstanding a conviction! This requires substantiation, and it shall be given. The other instances we have mentioned are so familiar to every reader of a daily paper, that quotation of examples is unnecessary. The recent assize reports will supply them in abundance, to anybody who will be at the trouble to examine them.

At the Quarter Sessions at Knutsford, on the 27th Nov. 1851, one James Blakeley was tried on a charge of assaulting a young woman, with intent, &c. No evidence was offered of any other assault. The jury acquitted him of the assault with intent, and found him guilty of a common assault. This was obviously a blunder; but the chairman of the sessions, a Mr. Mannering, immediately passed sentence of nine months' imprisonment, stating it to be for the "indecent" assault. Just two months afterwards, on the 27th of January last, a man was tried at the Clerkenwell Sessions for precisely the same offence, and under circumstances as nearly similar as could be. The same result followed. The jury acquitted him of the graver charge, and found him guilty of a common assault. In this case the judge was a lawyer, instead of that hermaphrodite class, a county magistrate. Mr. Serjeant Adams decided such a verdict to amount to an acquittal, and discharged the man.

Our criminal code is alike deficient in certainty of offence, certainty of prosecution, certainty of conviction, and certainty of punishment. The first, second, and fourth of these defects admit of remedy; and till that remedy is applied by a graduated classification of crime, by the appointment of a public prosecutor, and, more than all, by displacing the whole body of county justices, and transferring their criminal jurisdiction to responsible stipendiaries, educated in law, we cannot hope to obtain that restrictive efficacy upon the excessive indulgence of animal passions which ought to follow the administration of all punishment intended to be exemplary to others.

We have been betrayed into greater length than we intended ; and yet we feel that we have, after all, treated our subject more superficially than is consistent with its importance. We must plead the hacknied excuse, that a periodical essayist is limited to the opportunity of suggesting to others objects worthy of their attention, and the means whereby they may be accomplished. If our review of the progress of insanity and crime be correct, it is for the statesman to follow up the of the statist.

